# A new model of wheezing severity in young children using the validated ISAAC wheezing module: A latent variable approach with validation in independent cohorts

**DOI:** 10.1371/journal.pone.0194739

**Published:** 2018-04-17

**Authors:** Steven M. Brunwasser, Tebeb Gebretsadik, Diane R. Gold, Kedir N. Turi, Cosby A. Stone, Soma Datta, James E. Gern, Tina V. Hartert

**Affiliations:** 1 Vanderbilt University Medical Center, Department of Medicine, Division of Allergy, Pulmonary, and Critical Care Medicine, T-1218 Medical Center North, Nashville, TN, United States of America; 2 Vanderbilt University Medical Center, Department of Obstetrics and Gynecology, B-1118 MCN, Nashville, TN, United States of America; 3 Vanderbilt University Medical Center, Department of Biostatistics, West End, Nashville, TN, United States of America; 4 Channing Division of Network Medicine, Brigham and Women’s Hospital and Harvard Medical School, Department of Environmental Health, Boston, MA, United States of America; 5 Department of Environmental Health, Harvard T.H. Chan School of Public Health, Boston, MA, United States of America; 6 University of Wisconsin-Madison, School of Medicine and Public Health, Department of Pediatrics, K4/918 CSC, Madison, WI, United States of America; National Taiwan University College of Public Health, TAIWAN

## Abstract

**Background:**

The International Study of Asthma and Allergies in Children (ISAAC) Wheezing Module is commonly used to characterize pediatric asthma in epidemiological studies, including nearly all airway cohorts participating in the Environmental Influences on Child Health Outcomes (ECHO) consortium. However, there is no consensus model for operationalizing wheezing severity with this instrument in explanatory research studies. Severity is typically measured using coarsely-defined categorical variables, reducing power and potentially underestimating etiological associations. More precise measurement approaches could improve testing of etiological theories of wheezing illness.

**Methods:**

We evaluated a continuous latent variable model of pediatric wheezing severity based on four ISAAC Wheezing Module items. Analyses included subgroups of children from three independent cohorts whose parents reported past wheezing: infants ages 0–2 in the INSPIRE birth cohort study (Cohort 1; *n* = 657), 6-7-year-old North American children from Phase One of the ISAAC study (Cohort 2; *n* = 2,765), and 5-6-year-old children in the EHAAS birth cohort study (Cohort 3; *n* = 102). Models were estimated using structural equation modeling.

**Results:**

In all cohorts, covariance patterns implied by the latent variable model were consistent with the observed data, as indicated by non-significant χ^2^ goodness of fit tests (no evidence of model misspecification). Cohort 1 analyses showed that the latent factor structure was stable across time points and child sexes. In both cohorts 1 and 3, the latent wheezing severity variable was prospectively associated with wheeze-related clinical outcomes, including physician asthma diagnosis, acute corticosteroid use, and wheeze-related outpatient medical visits when adjusting for confounders

**Conclusion:**

We developed an easily applicable continuous latent variable model of pediatric wheezing severity based on items from the well-validated ISAAC Wheezing Module. This model prospectively associates with asthma morbidity, as demonstrated in two ECHO birth cohort studies, and provides a more statistically powerful method of testing etiologic hypotheses of childhood wheezing illness and asthma.

## Introduction

### Background

Asthma is among the most common and costly diseases affecting childhood [1,2]. Large epidemiological studies aimed at advancing understanding of the development and sequelae of asthma often rely on brief questionnaires that can be broadly implemented to characterize symptom severity [3,4]. Investigators for the International Study of Asthma and Allergies in Children (ISAAC) [3] developed and validated a research questionnaire assessing wheezing symptoms [3,5] that has since been used worldwide to assess the prevalence and etiology of asthma [4,6–10]. The ISAAC Wheezing Module (ISAAC-WM) includes eight items, four specifically focused on wheezing, either parent-reported for younger children or self-reported by older children [3]. The questionnaire and manual are available at http://isaac.auckland.ac.nz/story/methods/methods.php.

There is no consensus method for operationalizing wheezing severity using the ISAAC-WM. Respondents endorsing past-year wheezing complete three items assessing the frequency of wheezing episodes and negative sequelae in the past 12 months. Additionally, all respondents report on whether or not their child experienced exercise-induced wheezing [3]. Researchers typically stratify children into discrete severity groups based on frequency of wheezing episodes (item 3), wheeze-related sleep disturbance (item 4), wheeze-related speech disturbance (item 5) [7], exercise-induced wheezing (item 7), or a combination of these items [11].

Discrete severity variables are suboptimal in explanatory research as they artificially reduce variability, grouping individuals with truly differing severity levels into the same data bins [12]. This reduces power, likely resulting in underestimates of etiological associations and suboptimal theory testing [12,13]. A number of research groups have used exploratory techniques, like principal components [14,15] or latent factor approaches [16,17], to develop continuous asthma severity measures [18]. To our knowledge, however, no studies have tested the plausibility of these models using confirmatory analytic techniques. Additionally, we know of no studies, either exploratory or confirmatory, evaluating the ability of the ISAAC-WM to capture pediatric wheezing severity on a continuous scale for research purposes.

### Purpose

There are a number of large epidemiological studies evaluating pediatric wheezing illness using the ISAAC-WM [4,8,19,20], including nearly all airway cohorts participating in the Environmental Influences on Child Health Outcomes (ECHO) initiative [21]. ECHO is an NIH-funded consortium of previously established birth cohorts aimed at understanding the modifiable environmental etiologies of four major diseases, including asthma [21]. These studies contain rich data (e.g., early life exposures and wheeze-related clinical outcomes), providing opportunities to test etiological models of wheezing illness development and outcomes. To conduct strong tests of theory, however, it is critical to measure wheezing severity with optimal precision. The purpose of this study is to demonstrate how studies using the ISAAC-WM can use structural equation modeling (SEM) to estimate wheezing severity as a continuous latent variable. Using a continuous latent variable approach, rather than discrete severity variables, may lead to more precise measurement and stronger tests of etiological theory [22] in studies using the ISAAC-WM. This, in turn, could lead to improved understanding of pediatric wheezing illness and strengthen prevention and early intervention efforts.

SEM is a flexible multivariate statistical approach that supports precise specification of theoretical models and estimation of latent variables representing constructs that we cannot measure directly [22–25]. Latent *factors* capture shared variance among multiple measured variables (*factor indicators*) believed to have a common underlying cause [25,26]. In this study, we conceptualize wheezing illness as a latent (unobserved) variable that has measurable consequences (e.g., wheezing attacks, disturbed sleep). The four wheezing-focused ISAAC-WM items capture and quantify these observable manifestations of wheezing illness. If we assume that wheezing illness (the latent construct) is driving observed correlations among the ISAAC-WM wheezing items, then their shared variance can be used to estimate wheezing illness as a continuous latent variable. Modeling associations with well-specified latent variables generally results in increased statistical power [22,27].

We evaluated our proposed model of wheezing illness severity in three independent cohorts. Two of these cohorts are part of the Children’s Respiratory Research and Environment Workgroup (CREW) within the ECHO initiative [21], and the third is a publicly available data set from the ISAAC Phase I study [6]. Nearly all ECHO airway cohorts have used the ISAAC-WM, thus a more powerful approach to estimating severity in explanatory research studies with this instrument may be of broad interest. We address whether the proposed latent factor model of pediatric wheezing severity is:

1. consistent with observed data in all three cohorts
2. positively and concurrently associated with established markers of wheezing illness (convergent validity)
3. stable over repeated time points (longitudinal invariance) and across child sexes
4. positively and prospectively associated with wheeze-related clinical outcomes (predictive validity)

A valid latent variable approach to measuring wheezing severity with the ISAAC-WM may facilitate stronger tests of etiological theory and advancements in our understanding of pediatric wheezing illness.

## Materials and methods

### Data sources and measures

Table 1 provides demographic characteristics for each cohort.

**Table 1:** Participant characteristics for all cohorts.

	*n*	Proportion
**Cohort 1: INSPIRE (*n* = 657)**		
Maternal History of Asthma	154	0.23
Child RSV+ Molecular Diagnostic Test	66	0.10
Maternal Prenatal Cigarette Smoking (Any)	139	0.21
Medicaid Insurance at Enrollment	377	0.57
Child Cesarean Birth Delivery	224	0.34
Child Sex: Female	250	0.38
Mother Married at Enrollment	368	0.56
Child Race		
*African American*	167	0.25
*Asian*	10	0.02
*Native American/Native Alaskan*	6	0.01
*Native Hawaiian/Pacific Islander*	2	0.003
*White*	510	0.78
*Other Race*	37	0.06
Child Ethnicity: Hispanic/Latino	58	0.09
**Cohort 2: ISAAC Phase 1 (*n* = 2,765)**		
Child Sex: Female	1,193	0.43
**Cohort 3: EHAAS (*n* = 102)**		
Child Sex: Female	34	0.33
Child Race/Ethnicity		
*African American*	10	0.10
*Asian*	7	0.07
*Hispanic/Latino*	4	0.04
*White*	80	0.78
*Other Race*	1	0.01

#### Cohort 1. INSPIRE birth cohort study

The Infant Susceptibility to Pulmonary Infections and Asthma following RSV Exposure (INSPIRE) study is an ongoing population-based birth cohort that is part of the ECHO/CREW consortium, evaluating the role of early life respiratory infection in pediatric asthma [19,28]. The study enrolled term and otherwise healthy infants (*N* = 1,951) in Middle Tennessee. The Vanderbilt University Institutional Review Board (IRB) approved all study procedures. Wheezing symptoms are assessed annually using the parent-reported ISAAC-WM [19]. Although all participants complete the ISAAC-WM, given our focus on wheezing severity, we included only children whose parents reported wheezing in the first two years of life (*n* = 657).

At both the one- and two-year assessments, parents reported whether or not their children used asthma medications and/or had been hospitalized for respiratory illnesses in the prior year. With regard to asthma medications, parents were asked explicitly if their children had been treated with any of the following: Budesonide, Fluticasone, Beclomethasone, Montelukast, Albuterol, Budesonide-Salmeterol, Fluticasone-Salmeterol, Prednisone/Prednisolone, Dexamethasone, or any other asthma medications. At the year-three assessment, parents reported several wheeze-related clinical outcomes: physician diagnosis of asthma at any point in life (*Absent* or *Present*), past-year treatment of asthma symptoms with any form of corticosteroid (*Never*, *1–3 times*, *4+ times*), and past-year frequency of wheeze-related healthcare visits (*None*, *1–3*, *4+*). When asking parents about corticosteroid use, no distinction was made between parenteral steroids or oral corticosteroids in the questioning.

#### Cohort 2. ISAAC Phase 1 study

Cohort 2 analyses used publicly available data from the ISAAC Study Phase I (http://isaac.auckland.ac.nz/phases/phaseone/results/resultsIndv.php) to evaluate whether the covariance patterns implied by the latent wheezing severity model established in Cohort 1 were consistent with observed covariance patterns in an independent dataset. Detailed study procedures are available in prior publications [3,5]. ISAAC Phase I included two cohorts of children (ages 6–7 and ages 13–14) from 38 countries. We limited analyses to 6-7-year-old children from the three North American countries: Canada, U.S.A., and Barbados. This was felt to be most representative of the ECHO/CREW consortium sites. Analyses included the subset of children who, by parent report, had ever had wheezing episodes (*n* = 2,765).

#### Cohort 3. EHAAS birth cohort

Cohort 3 analyses evaluated whether the latent wheezing severity model established with Cohort 1 was consistent with observed data patterns in the Epidemiology of Home Allergens and Asthma Study (EHAAS) study, and whether the latent factor was prospectively associated with wheezing morbidity. EHAAS is a prospective birth cohort study that is also part of the ECHO/CREW consortium (*N* = 505) [20,29–31]. Families from a Boston hospital were eligible if the biological mother was 18+ years old at enrollment, at least one biological parent had a history of allergies or asthma, infants were born term without major congenital anomalies and were not admitted to the neonatal intensive care unit. Study procedures were approved by the Brigham and Women’s Hospital IRB. We evaluated ISAAC-WM data collected when children were 5 and 6 years old. Analyses were limited to the subsample of children whose parents reported wheezing episodes at some point during the two-year follow-up window (*n* = 102). Clinical outcomes at age 7 included whether or not the child had: (1) a physician diagnosis of asthma, (2) any prior-year wheezing-related medical visits, and (3) any prior-year wheezing-related urgent visits to a healthcare provider’s office or emergency department.

### Statistical analyses

**Fig 1:**
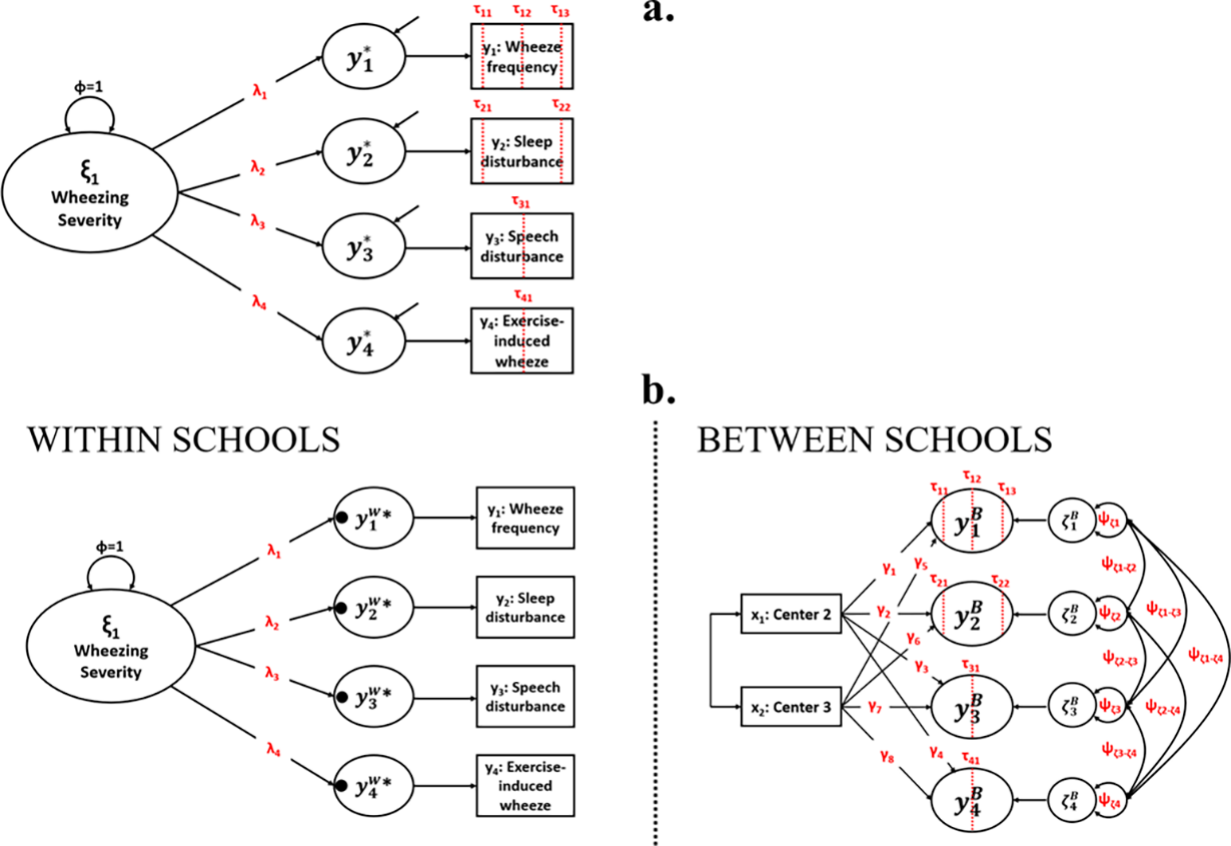
Latent variable model of wheezing illness severity. Panel *a* shows the latent wheezing severity model used in cohorts 1 and 3. The severity of wheezing illness is estimated as a unidimensional latent variable (η_1_) with four reflective ordinal indicators: wheezing episode frequency (y_1_), frequency of wheeze-related sleep disturbance (y_2_), wheeze-related speech disturbance (y_3_), and exercise-induced wheeze (y_4_). The ordinal indicators are presumed to be coarse measurements of underlying continuous variables (y_1_*-y_4_*). Panel *b* shows the multilevel wheezing severity model used in the Cohort 2 analyses. The within-schools level of the model is identical to panel *a*. The between-schools level of the model accounts for non-independence due to clustering within schools and study sites. Estimated parameters are depicted in red.

Structural equation modeling (SEM) was the primary analytic strategy with parameters estimated via robust weighted least squares [32]. Fig 1A shows the hypothesized latent wheezing severity model. Associations between the latent wheezing severity variable and the ordinal ISAAC-WM severity items were estimated using a latent response variable framework [22,32,33], in which ordered categorical outcomes (y_1,_ y_2,_ y_3,_ y_4_) are conceptualized as coarse approximations of underlying continuous variables (y_1_*, y_2_*, y_3_*, y_4_*). The models used probit regressions to estimate *c*-1 thresholds (τ) for ordinal outcomes, where *c* is the number of ordered levels, dividing the underlying continuous distributions into discrete categories [32]. Analyses were conducted using the lavaan package version 0.5–22 [34] in R [35] and Mplus version 7.4 [36]. Missing data in the response variables were presumed to be missing at random conditional on the model covariates [37].

The ISAAC Phase 1 data (Cohort 2) had a complex structure with children nested within schools (*j* = 282) within study sites (*k* = 3). We used multilevel SEM [38,39] to evaluate the latent wheezing severity factor model while accounting for clustering (dependence) within schools (Fig 1B). On the within-school level, the wheezing severity model was identical to the model specified in Cohort 1. On the between-schools level, we specified a saturated model where all indicator variances and covariances were freely estimated and the indicators were regressed on dummy variables coding study site. This approach accounts for between-school and between-site variability, but presumes that wheezing severity is a meaningful construct only on the within-schools level of analysis; that is, wheezing severity is only intended to be measured as a child-level characteristic, not a school-level characteristic [40].

In the Cohort 3 analyses, to avoid sparse cells for the ISAAC-WM indicators, data for each of the ISAAC-WM items were aggregated across the age-5 and age-6 assessments (Table 2). These aggregated items were used in all models.

**Table 2:** Cohort 3 EHAAS study: Derived wheezing severity items characterizing the severity of wheeze across the 60- and 72-month assessments combined.

ISAAC-WM WHEEZING ITEM	DERIVED SEVERITY LEVELS COMBINING ACROSS THE 60- AND 72-MONTH ASSESSSMENTS
**Item 3.** Number of wheezing attacks in past 12 months	Very infrequent (value = 0) • *None* (60-Month) and *1–3 attacks* (72-Month) • *1–3 attacks* (60-Month) and *None* (72-Month) Infrequent (value = 1) • *1–3 attacks* (60-Month) and *1–3 attacks* (72-Month) • *None* (60-Month) and *4–12 attacks* (72-Month) • *4–12 attacks* (60-Month) and *None* (72-Month) Frequent (value = 2) • *1–3 attacks* (60-Month) and *4–12 attacks* (72-Month) • *4–12 attacks* (60-Month) and *1–3 attacks* (72-Month) • *None* (60-Month) and *13+ attacks* (72-Month) • *13+ attacks* (60-Month) and *None* (72-Month) Very frequent (value = 3) • *4–12 attacks* (60-Month) and *4–12 attacks* (72-Month) • *4–12 attacks* (60-Month) and *13+ attacks* (72-Month) • *13+ attacks* (60-Month) and *4–12 attacks* (72-Month) • *13+ attacks* (60-Month) and *13+ attacks* (72-Month)
**Item 4.** Child’s sleep disturbed due to wheezing	Never (value = 0) • *Never* (60-Month) and *Never* (72-Month) Infrequent (value = 1) • *Never* (60-Month) and *< 1 night/week* (72-Month) • *< 1 night/week* (60-Month) and *Never* (72-Month) Frequent (value = 2) • *Never* (60-Month) and *1+ nights/week* (72-Month) • *1+ nights/week* (60-Month) and *Never* (72-Month) • *<1 night/week* (60-Month) and *<1 night/week* (72-Month) • *<1 night/week* (60-Month) and *1+ nights/week* (72-Month) • *1+ nights/week* (60-Month) and *<1 night/week* (72-Month) • *1+ nights/week* (60-Month) and *1+ nights/week* (72-Month)
**Item 5.** Wheezing ever severe enough to child’s speech to only one or two words at a time between breaths	Absent at both assessments (value = 0) Present at either or both assessments (value = 1)
**Item 7.** Wheezing or whistling in chest during or after exercise	Absent at both assessments (value = 0) Present at either or both assessments (value = 1)

#### Assessing model adequacy

In all three cohorts, the degree of discrepancy between proposed models and observed data was assessed using a mean- and variance-adjusted χ^2^ Goodness of Fit (χ^2^_GOF_) test [32,37,41,42]. Significant χ^2^_GOF_ values lead to a rejection of the null hypothesis of a perfect correspondence between the model and the observed data [22]. We reported two additional indices of model adequacy: the root mean error of approximation (RMSEA) [43,44] and comparative fit index (CFI) [45]. The RMSEA provides an estimate of the amount of misspecification per model degree of freedom with a 90% confidence interval (CI), whereas the CFI compares the hypothesized model to a simpler baseline (null) model, penalizing the hypothesized model for each estimated parameter [46]. Although there are no perfect cutoff values for these indices [47], RMSEA values < .05 and CFI values ≥ .95 are generally considered to be indicative of a desirable model [48].

It is noteworthy that, owing to the relatively small sample size, Study 3 models were underpowered to detect significant model misspecification with the χ^2^_GOF_ test. Consequently, we relied on data from studies 1 and 2 to assess whether our proposed latent wheezing severity model was consistent with the observed data, whereas Study 3 analyses were focused on evaluating whether the latent wheezing severity variable was prospectively associated with established markers of wheezing illness severity (predictive validity).

#### Convergent validity

Using Cohort 1 data at both the year-one and year-two assessments, we specified models evaluating concurrent associations between the latent wheezing severity factor and parent-reported markers of wheezing illness: wheezing medication use (*Any* vs. *None*) and respiratory hospitalizations (*Any* vs. *None*). These associations were adjusted for seven potential confounders representing characteristics that have been linked to asthma risk in prior studies [49]: maternal asthma history [50] (*Absent* vs. *Present*), maternal prenatal cigarette use [51] (*None* vs. *Any*), maternal marital status [52] (*Married* vs. *Single*), insurance at enrollment as a proxy for socioeconomic status [53] (*Private* vs. *Medicaid*), birth method [54] (*Vaginal* vs. *Cesarean*), child sex [55], and child race [55] (African American, all other race groups, and white race [reference group]). Both the markers of wheezing illness severity (validity outcomes) and the latent wheezing factor were regressed on these seven asthma risk factors.

#### Latent factor stability

Using Cohort 1 data, we evaluated whether our latent wheezing severity model was stable across the one- and two-year follow-ups using procedures described in Liu et al. (2016) [56]. An unstable factor structure indicates that the latent construct cannot be measured reliably in the same way across repeated assessments, making it challenging to interpret change over time [56–58]. Four competing models, representing differing degrees of instability, were specified and compared using scaled χ^2^ likelihood ratio tests [59]. Significant χ^2^ tests are *not* desirable in this context because they indicate that models allowing for instability in the factor structure fit better than more restrictive models that assume stability. The most desirable model allows mean levels of wheezing severity (the latent factor) to change over time, but holds the measurement of wheezing severity (factor loadings, thresholds, and residual variances) constant over time. In all models, the latent wheezing factor was regressed on the seven asthma risk factors described above, with associations between risk factors and the latent factor permitted to vary freely across time points. We used similar procedures [60] to test whether the latent factor measurement was consistent across sexes (female vs. male children) [61].

#### Predictive validity

Using Cohort 1 data, we specified longitudinal models evaluating whether the latent wheezing illness factor at the two-year assessment was prospectively associated with year-three clinical outcomes, including physician asthma diagnosis, acute corticosteroid use, and wheeze-related outpatient visits. These prospective associations were adjusted for all seven asthma risk factors described above. Using Cohort 3 data, we evaluated whether the latent wheezing severity variable measured at ages 5–6 was prospectively associated with wheeze-related outcomes at age 7, adjusting for child sex and race.

#### Comparison to discrete severity models

Using data from Cohort 1, we gauged whether the latent factor approach to estimating wheezing severity as a continuous construct had greater predictive utility relative to a more traditional approach with a discrete severity variable. To accomplish this, we first derived a five-level ordinal wheezing severity variable indicating the number of ISAAC-WM wheezing items endorsed (i.e., present to some degree) at the year-two assessment (*None*, *1*, *2*, *3*, *4*). We then reran the latent variable predictive validity models described in the prior section, using the same covariates and estimation procedures, but replacing the latent wheezing severity factor with the ordinal severity variable. This allowed us to evaluate whether the latent variable modeling approach yielded stronger associations with the future clinical outcomes (physician asthma diagnosis, acute corticosteroid use, and wheeze-related outpatient visits) compared to the more traditional discrete severity approach.

## Results

Table 3 provides descriptive statistics for the ISAAC-WM severity items for all three cohorts. Separate online appendixes for Cohort 1 (S1 Appendix), Cohort 2 (S2 Appendix), and Cohort 3 (S3 Appendix) are available providing model code. For cohorts 1 and 3, we provide sufficient summary statistics (covariance matrixes, weighted least squares weight vector, asymptotic variance matrix, and sample size) to allow others to reproduce our models (S1 File).

**Table 3:** Descriptive statistics for ISAAC-wheezing module severity items.

	LEVELS	COUNT	PROP	COUNT	PROP
**Cohort 1: INSPIRE**		**1st Year of Life**		**2nd Year of Life**	
*# of Wheeze Episodes*	Never	205	0.31	156	0.24
	1 to 3	328	0.50	274	0.42
	4 to 12	73	0.11	76	0.12
	13+	28	0.04	17	0.03
	Missing	23	0.04	134	0.20
*Wheeze Disturbs Sleep*	Never	455	0.69	336	0.51
	< 1 night/week	83	0.13	93	0.14
	1+ nights/week	96	0.15	94	0.14
	Missing	23	0.04	134	0.20
*Wheeze Disturbs Speech*	Present	116	0.18	100	0.15
	Missing	24	0.04	135	0.21
*Exercise-Induced Wheeze*	Present	67	0.10	76	0.12
	Missing	14	0.02	33	0.05
**Cohort 2: ISAAC Phase 1**					
*# of Wheeze Episodes*	Never	1,173	0.42		
	1 to 3	1,074	0.39		
	4 to 12	330	0.12		
	13+	107	0.04		
	Missing	81	0.03		
*Wheeze Disturbs Sleep*	Never	1,793	0.65		
	< 1 night/week	641	0.23		
	1+ nights/week	228	0.08		
	Missing	103	0.04		
*Wheeze Disturbs Speech*	Present	259	0.09		
	Missing	91	0.03		
*Exercise-Induced Wheeze*	Present	714	0.26		
	Missing	119	0.04		
**Cohort 3: EHAAS**					
*# of Wheeze Episodes*	Very Infrequent	31	0.30		
	Infrequent	39	0.38		
	Frequent	12	0.12		
	Very frequent	16	0.16		
	Missing	4	0.04		
*Wheeze Disturbs Sleep*	Never	55	0.54		
	Infrequent	23	0.23		
	Frequent	21	0.21		
	Missing	3	0.03		
*Wheeze Disturbs Speech*	Present	11	0.11		
	Missing	3	0.03		
*Exercise-Induced Wheeze*	Present	28	0.27		
	Missing	1	0.01		

PROP = proportion of the total sample.

### Model fit

Table 4 provides information about model adequacy for all models. Model numbers are provided in the table and are referenced in the text below.

**Table 4:** Indices of model adequacy.

	$\chi_{GOF}^{2}$			RMSEA		CFI	Model Description
**Cohort 1: INSPIRE Birth Cohort**							
	Est	*df*	*p*	Est	90% CI	Est	
Model 1.1	2.35	2	.31	.02	.00, .08	1.0	Y1: Wheeze severity model
Model 1.2	3.76	2	.15	.04	.00, .10	1.0	Y2: Wheeze severity model
Model 1.3	27.19	29	.56	.00	.00, .03	1.0	Y1: Concurrent association with respiratory hospital visit
Model 1.4	31.08	29	.36	.01	.00, .03	.97	Y2: Concurrent association with respiratory hospital visit
Model 1.5	26.75	29	.59	.00	.00, .03	1.0	Y1: Concurrent association with asthma medication
Model 1.6	29.47	29	.44	.01	.00, .03	1.0	Y2: Concurrent association with asthma medication
Model 1.7	29.48	29	.44	.01	.00, .03	.99	Prospective associations with Y3 asthma diagnosis
Model 1.8	29.28	29	.45	.00	.00, .03	1.0	Prospective associations with Y3 corticosteroids
Model 1.9	29.01	29	.46	.00	.00, .03	1.0	Prospective associations with Y3 wheeze medical visits
**Cohort 2: ISAAC Phase 1**							
Model 2.1	0.48	2	.79	.02	NA	1.0	Wheeze severity model
Model 2.2	7.20	5	.21	.00	NA	1.0	Regression of wheeze severity factor on child sex
**Cohort 3: EHAAS Birth Cohort**							
Model 3.1	0.83	2	.66	.00	.00, .15	1.0	Wheeze severity model
Model 3.2	4.00	8	.86	.00	.00, .06	1.0	Regression of wheeze severity on child sex and race
Model 3.3	7.01	11	.80	.00	.00, .07	1.0	Predictive validity: 84-month asthma dx.
Model 3.4	7.22	11	.78	.00	.00, .07	1.0	Predictive validity: 84-month wheeze visits
Model 3.5	6.05	11	.87	.00	.00, .05	1.0	Predictive validity: 84-month ER visits

$\chi_{GOF}^{2}$ = model chi-square goodness of fit test; RMSEA = root mean error of approximation; CFI = Comparative Fit Index; Y1-Y3 = Year 1 –Year 3; NA = RMSEA confidence intervals were not available for multilevel SEM models.

#### Cohort 1. INSPIRE birth cohort

The unidimensional latent factor model of wheezing illness severity (Fig 1A) provided a close fit to the observed data at both the one- and two-year assessments, as indicated by non-significant χ^2^_GOF_ tests (Table 4, models 1.1 and 1.2). At both assessments, there were strong positive associations between the latent wheezing factor and the ISAAC-WM wheezing indicators (S1 Fig). Consistent with prior research using a different instrument [16], information curves showed that levels of wheezing illness severity factor could be estimated with greatest precision at moderate to high levels of illness severity: The latent factor was less informative for children with mild wheezing symptoms (S2 Fig).

#### Cohort 2. ISAAC Phase 1

Consistent with Cohort 1 findings, the multilevel SEM model evaluating the wheezing severity as a unidimensional construct yielded a non-significant χ^2^_GOF_ test (Table 4, Model 2.1), indicating a close fit to the data. Associations between the latent factor and all four indicators were positive and significant on the within-school level of analysis.

#### Cohort 3. EHAAS birth cohort

Consistent with findings from cohorts 1 and 2, the hypothesized unidimensional factor model yielded a non-significant $\chi_{GOF}^{2}$ test (Table 4, Model 3.1). Thus, there was no evidence of model misspecification. This finding should be interpreted cautiously, however, as the model was underpowered to detect a significant discrepancy between the model and data because of the relatively small sample size in the Cohort 3 analyses.

### Convergent validity

**Fig 2:**
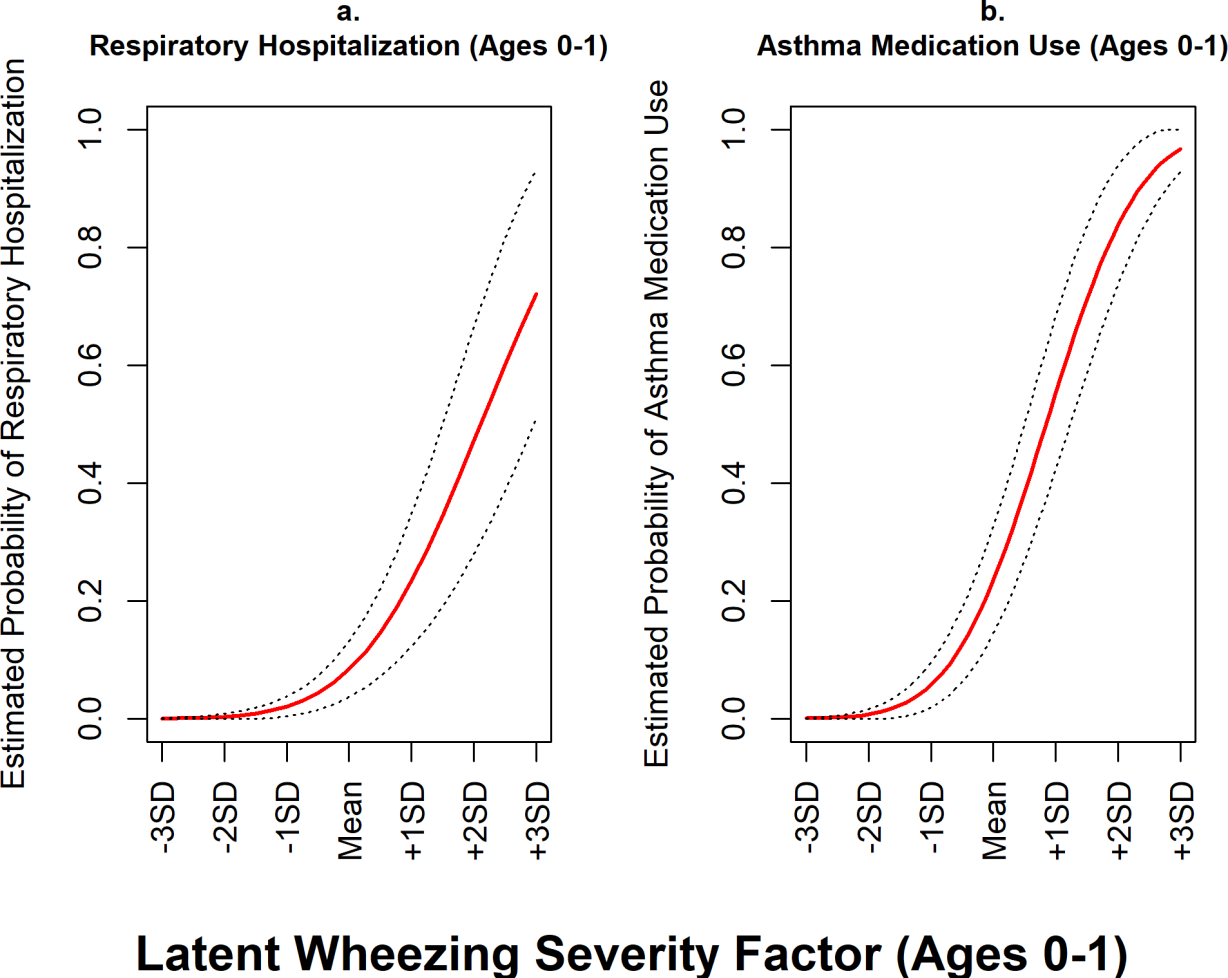
Cohort 1: Concurrent associations between the latent wheezing severity factor and markers of wheezing illness in year-one. This figure shows estimated probabilities of having at least one respiratory hospitalization (panel *a;* model 1.3) and using asthma medication (panel *b*; model 1.5) against levels of the latent wheezing severity variable in the first year of life. As wheezing severity increases so does the estimated probability of respiratory hospitalization and medication use. These models held all covariates constant at their median values. Dotted lines represent 95% confidence intervals for estimated probability estimates.

Using Cohort 1 data at both the one- and two-year assessments, the wheezing illness factor was positively associated with hospitalization for respiratory illness (Table 5, models 1.3 and 1.4) and asthma medication use (Table 5, models 1.5 and 1.6) adjusting for covariates. Fig 2 shows the magnitude of these concurrent associations at year-one.

**Table 5:** Cohort 1: Associations between the latent wheezing illness severity factors at both year-1 and year-2 and established markers of wheezing illness severity.

LATENT FACTOR	MODEL	EST	95% CI	
*Marker of Wheezing Illness Severity*	#	β^a^	Lower	Upper
**Year 1 Wheezing Illness Severity (Ages 0–1)**				
*Ages 0–1 Wheeze -Related Hospitalizations*	1.3	0.68	0.51	0.86
*Ages 0–1 Asthma Medication Use*	1.5	0.87	0.72	1.01
**Year 2 Wheezing Illness Severity (Ages 1–2)**				
*Ages 1–2 Wheeze -Related Hospitalizations*	1.4	0.54	0.35	0.72
*Ages 1–2 Asthma Medication Use*	1.6	0.90	0.77	1.03
*Ages 2–3 Asthma Diagnosis*	1.7	0.71	0.53	0.88
*Ages 2–3 Acute Corticosteroid Use*	1.8	0.63	0.49	0.76
*Ages 2–3 Wheeze-Related Medical Visits*	1.9	0.64	0.48	0.79

^a^Linear regression coefficient estimating the association between the latent wheezing illness severity factor and the continuous latent variable (y*) underlying the observed categorical outcome variable (y) that is an established marker of wheezing severity.

### Latent factor stability

Using Cohort 1 data, there was no evidence from likelihood ratio χ^2^ tests (*p* values ≥ .20) that imposing invariance restrictions on the loadings, thresholds, or unique variances across time points worsened model fit (Table 6). Thus, there was no evidence of temporal instability, allowing for meaningful evaluation of change in the latent factor. Additionally, there was no evidence of latent factor instability across child sex at either year-one or year-two (*p* values ≥ .25; Table 6), indicating that the same latent wheezing severity model was reasonable for girls and boys

**Table 6:** Cohort 1: Comparison of models testing stability in the latent wheezing severity factor across time and across child sex.

	Measurement Parameters			$\chi_{GOF}^{2}$			Model Comparison		
	Factor Loadings	Indicator Thresholds	Residual Variances	Est.	*df*	*p*	Models	$\chi_{DIFF}^{2}$	*p*
Models Evaluating Measurement Stability Across One- and Two-Year Assessments									
1	Free	Free	Free	61.97	62	.48	-	-	-
2	Constant	Free	Free	64.52	65	.49	2 vs 1	0.22	.97
3	Constant	Constant	Free	68.19	68	.47	3 vs 2	4.10	.20
4	Constant	Constant	Constant	69.58	72	.56	4 vs 3	0.92	.88
Models Evaluating Measurement Stability Across Child Sex (Female vs. Male) at Year-One									
5	Free	Free	Free	46.31	46	.46	-	-	-
6	Constant	Free	Free	49.64	49	.45	6 vs 5	2.97	.25
7	Constant	Constant	Free	51.45	51	.46	7 vs 6	0.63	.60
8	Constant	Constant	Constant	52.69	55	.56	8 vs 7	0.68	.85
Models Evaluating Measurement Stability Across Child Sex (Female vs. Male) at Year-Two									
9	Free	Free	Free	38.95	46	.76	-	-	-
10	Constant	Free	Free	41.01	49	.78	10 vs 9	0.72	.72
11	Constant	Constant	Free	42.95	51	.78	11 vs 10	1.24	.37
12	Constant	Constant	Constant	47.83	55	.74	12 vs 11	2.70	.37

$\chi_{GOF}^{2}$ = chi-square goodness of fit statistic evaluating whether the model is consistent with the observed data; $\chi_{DIFF}^{2}$ = chi-square test comparing the fit of competing nested models; Free = parameter was allowed to vary freely across groups (i.e., time points or child sex); Constant = parameter was constrained to be equal across groups (i.e., time points or child sex).

Consistent with prior research [62], male children scored higher on average than female children on the wheezing illness severity factor in all three cohorts. In Cohort 1 analyses, male children scored 0.20 (95% CI [0.02, 0.45]) standard deviations (*SD*s) higher (more severe symptoms) than females on the latent factor in the second year of life. Similarly, in cohorts 2 (model 2.2) and 3 (model 3.2), male children scored 0.09 *SD*s (95% CI [0.003, 0.18]) and 0.68 *SD*s (95% CI [0.01, 1.35]) higher on wheezing severity than females, respectively.

### Predictive validity

**Fig 3:**
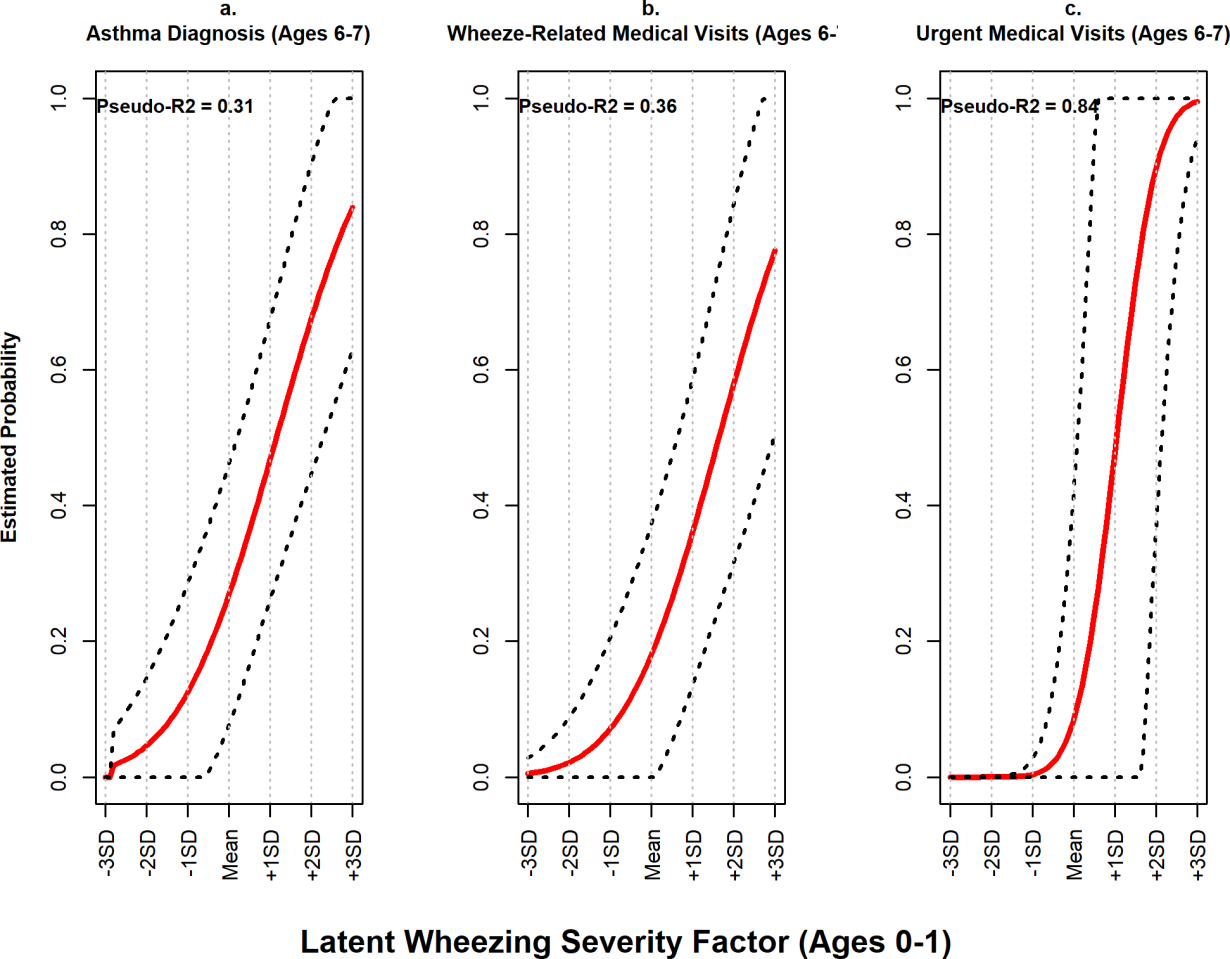
Cohort 3: Prospective associations between wheezing illness severity and wheeze-related morbidity outcomes. In the EHAAS birth cohort study, there were strong prospective associations between the latent wheezing illness severity factor representing wheezing illness severity from 60–72 months and three asthma morbidity outcomes at the 84-month follow-up: physician diagnosis of asthma (panel *a*; model 3.3), any asthma medical visits (panel *b*; model 3.4), and urgent asthma/wheeze-related medical visits to either a doctor’s office or the emergency department (panel *c*; model 3.5). These models held all covariates constant at their median values. Dotted lines represent 95% confidence intervals. Pseudo-R^2^ values represent the approximate proportion of variance in the outcome accounted for by the predictors.

In Cohort 1 longitudinal models, the severity of wheezing illness in the second year of life was uniquely and prospectively associated with year-three physician asthma diagnosis (model 1.7), acute corticosteroid use (model 1.8), and wheeze-related outpatient visits (model 1.9), adjusting for covariates (Table 5). Using Cohort 3 data, adjusting for covariates, the latent wheezing illness factor representing wheezing severity measured at ages 5–6 was prospectively associated with all clinical outcomes measured at age 7 (Fig 3): physician asthma diagnosis (model 3.3; β = 0.52, 95% CI [0.22, 0.82]), wheeze -related medical visits (model 3.4; β = 0.58, 95% CI [0.20, 0.96]), and urgent wheeze-related medical visits (model 3.5; β = 0.82, 95% CI [0.41, 1.24]).

### Comparison with discrete wheezing severity model

**Fig 4:**
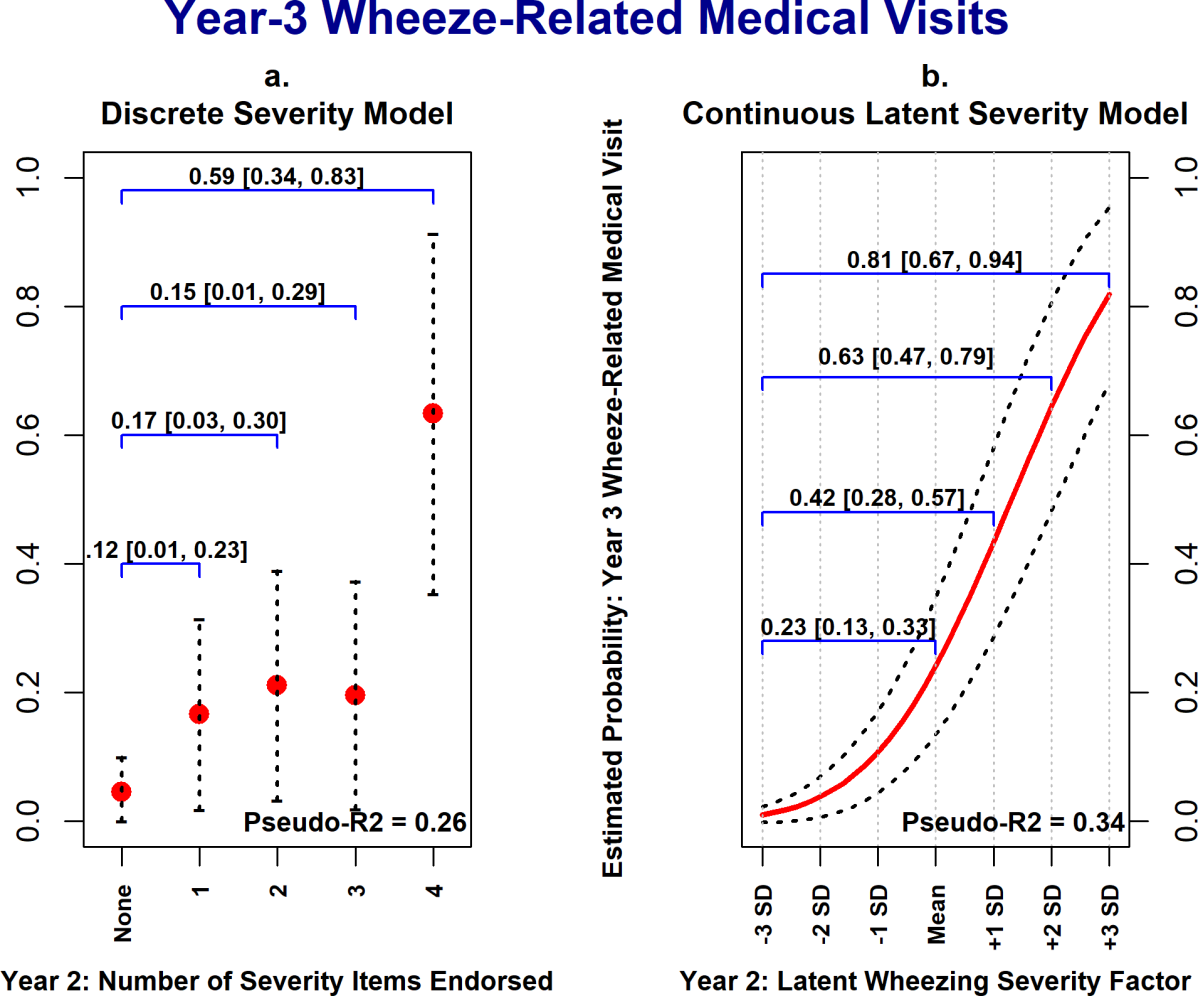
Cohort 1: Estimated probability of year-three wheeze-related medical visits as a function of wheezing severity in year-2. These plots show the strength of associations between year-2 wheezing severity (x-axis) and year-3 wheeze-related medical visits (y-axis), with all covariates held constant at their median values. Panels *a* shows estimated probabilities of corticosteroid treatment being present in the third year of life as a function of the discrete severity exposure variable; whereas panel *b* shows estimated probabilities vs. the latent continuous severity factor. In both models, as year-2 wheezing severity increases, so does the estimated probability of acute corticosteroid treatment, though the range of estimated probabilities is larger in the latent severity model. Values and 95% confidence intervals above the blue brackets show the increase in the estimated probabilities for a given increase in wheezing severity. Dotted lines represent 95% confidence intervals for estimated probability estimates.

Using Cohort 1 data, the discrete wheezing severity variable coding the number of ISAAC-WM severity items endorsed at the year-two assessment was significantly and positively associated with year-three asthma diagnosis, acute corticosteroid use, and wheeze/asthma-related outpatient visits. Thus, the discrete severity exposure variable had utility in predicting future wheezing morbidity. However, Fig 4 illustrates the potential benefits of modeling wheezing severity as a latent continuous variable in explanatory models. Fig 4A plots the estimated probability of a child having at least one wheeze-related medical visit in the third year of life against the year-2 discrete wheezing severity variable, adjusting for covariates. As the number of ISAAC-WM severity items endorsed increased in the second year of life (x-axis) the estimated probability of a child having a wheeze-related medical visit in the third year of life (y-axis) increased. In the discrete severity model, however, there are only five possible values of the estimated probability of year-three wheeze-related medical visits, ranging from 0.05 to 0.63, corresponding to the five levels of the discrete severity variable.

**Fig 5:**
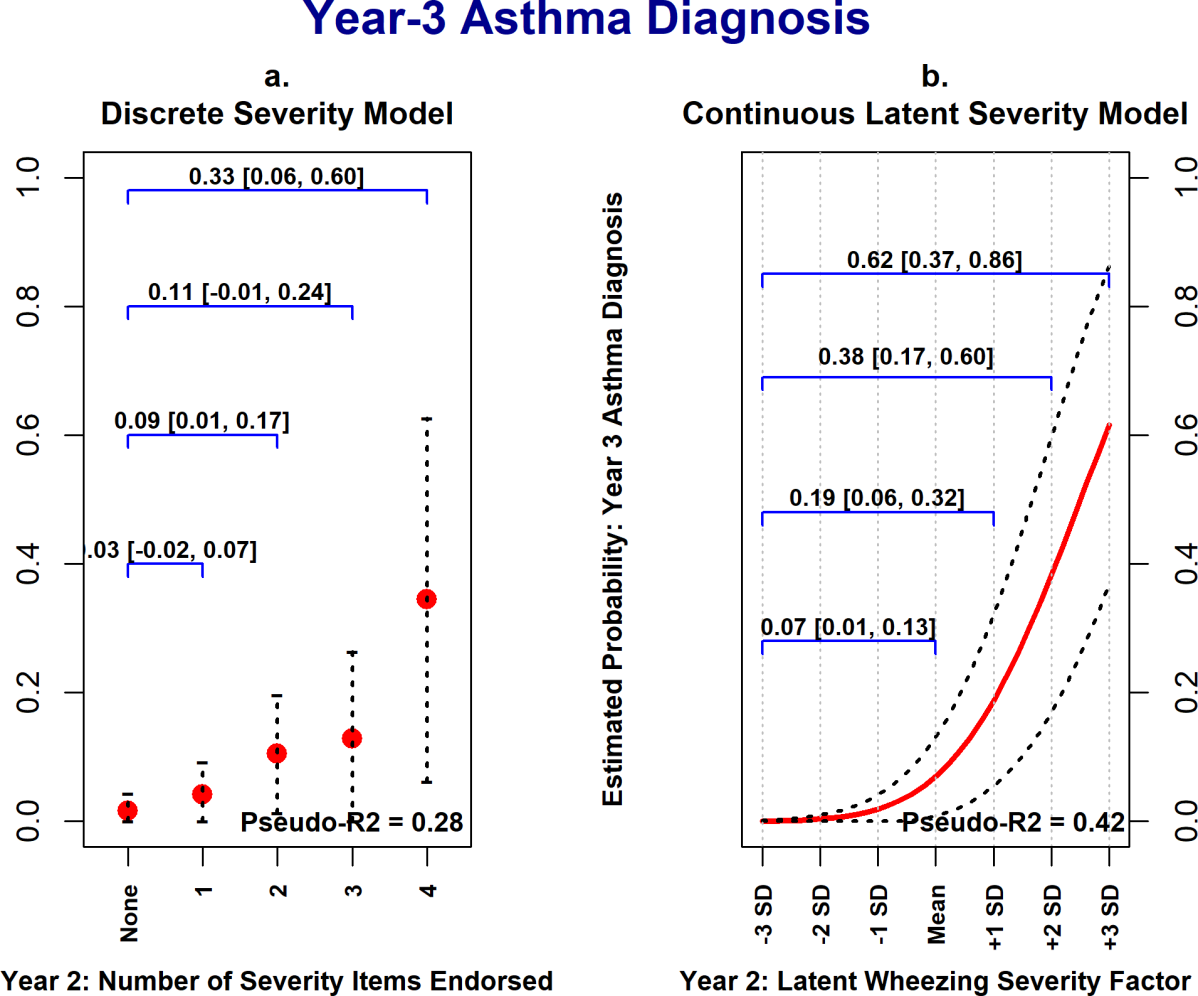
Cohort 1: Year-3 physician asthma diagnosis as a function of wheezing severity in year-2. These plots show the strength of associations between year-2 wheezing severity (x-axis) and year-3 physician asthma diagnosis (y-axis), with all covariates held constant at their median values. Panels *a* shows estimated probabilities of a physician asthma diagnosis being present in the third year of life as a function of the discrete severity exposure variable; whereas panel *b* shows estimated probabilities vs. the latent continuous severity factor. In both models, as year-2 wheezing severity increases, so does the estimated probability of a physician asthma diagnosis being present, though the range of estimated probabilities is larger in the latent severity model. Values and 95% confidence intervals above the blue brackets show the expected increase in the estimated probabilities for a given increase in wheezing severity. Dotted lines represent 95% confidence intervals for estimated probability estimates.

**Fig 6:**
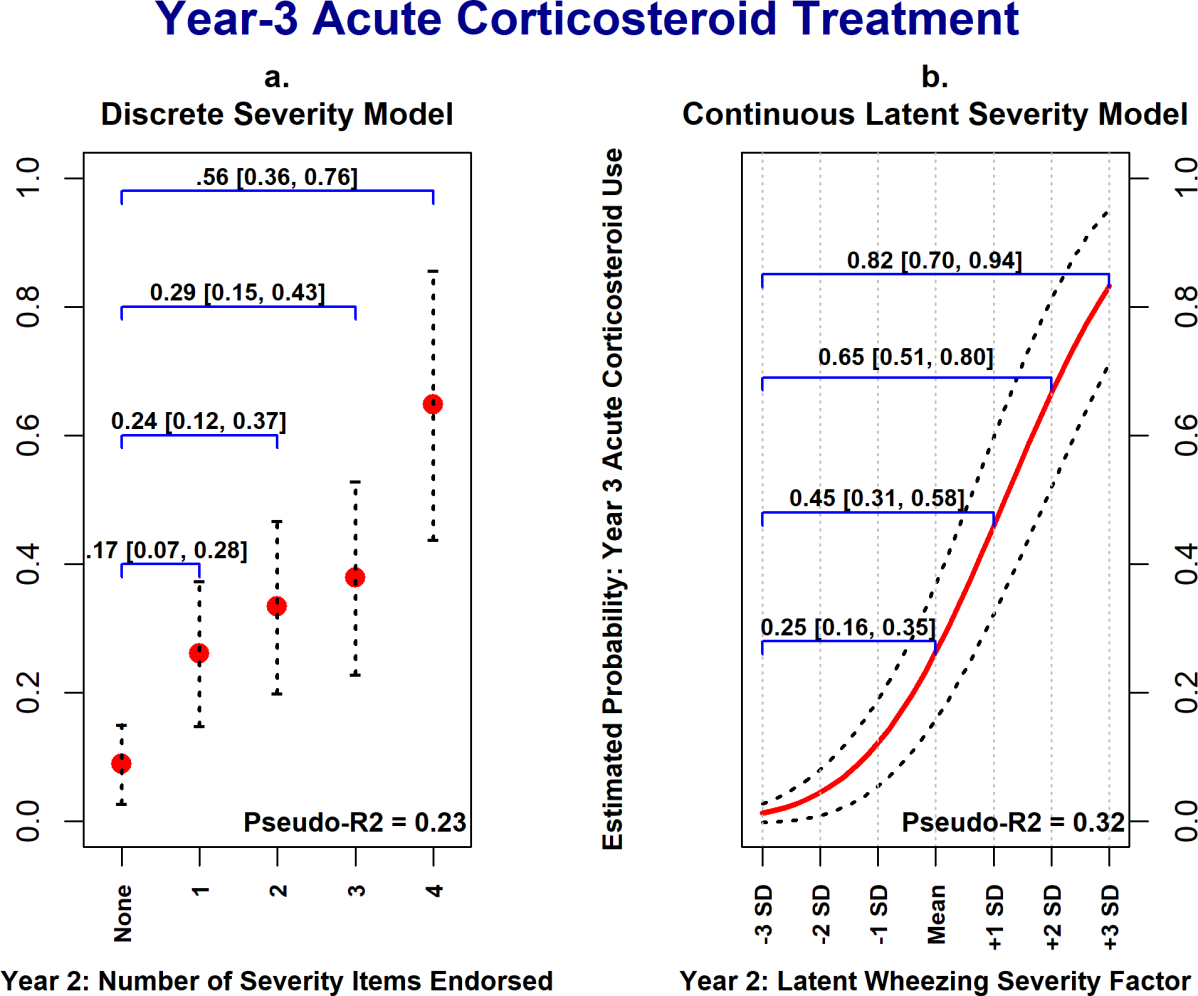
Cohort 1: Year-3 estimated probability of acute corticosteroid treatment as a function of wheezing severity in year-2. These plots show the strength of associations between year-2 wheezing severity (x-axis) and year-3 acute corticosteroid treatment (y-axis), with all covariates held constant at their median values. Panels *a* shows estimated probabilities of corticosteroid treatment being present in the third year of life as a function of the discrete severity exposure variable; whereas panel *b* shows estimated probabilities vs. the latent continuous severity factor. In both models, as year-2 wheezing severity increases, so does the estimated probability of acute corticosteroid treatment, though the range of estimated probabilities is larger in the latent severity model. Values and 95% confidence intervals above the blue brackets show the increase in the estimated probabilities for a given increase in wheezing severity. Dotted lines represent 95% confidence intervals for estimated probability estimates.

In contrast, Fig 4B shows the estimated probabilities of year-3 wheeze-related medical visits as a function of the latent wheezing severity factor, with estimated probabilities following a smooth curve ranging from 0.01 (at -3 *SD*s) to 0.82 (at +3 *SD*s). Going from the lowest level of the discrete wheezing severity variable to the highest results in a 0.59 (95% CI [0.34, 0.83]) increase in the estimated probability of year-3 wheeze-related medical visits; whereas going from 3 *SD*s below the mean to 3 *SD*s above the mean on the latent severity variable results in an increase in the estimated probability of 0.81, 95% CI [0.67, 0.94]. Similar patterns were observed for year-3 physician asthma diagnosis (Fig 5) and acute corticosteroid treatment (Fig 6). Also, the degree of uncertainty around the estimated probabilities was smaller when using the latent wheezing severity model. This is consistent with our central premise that a latent severity model can provide improved estimation of severity, resulting in stronger associations with clinical outcomes.

## Discussion

### Summary of key findings

The findings from three independent studies support the utility of a unidimensional latent variable approach to measuring pediatric wheezing illness severity based on items from the ISAAC-WM in explanatory research studies. We found:

1. a close correspondence between the latent wheezing severity model and the observed data (cohorts 1 and 2);
2. concurrent (Cohort 1) and prospective (cohorts 1 and 3) associations with wheeze-related clinical outcomes;
3. stability in the latent wheezing severity factor structure across time points and child sex (Cohort 1);
4. stronger prospective associations with wheezing outcomes compared to a discrete severity variable (Cohort 1).

### Interpretation and implications

Wheezing is the hallmark of asthma and an indicator of asthma control [63,64]. The ISAAC questionnaire is a validated instrument used in epidemiologic studies worldwide [3,4]. The use of wheezing questions to assess disease severity in etiological research is highly desirable, particularly for the new ECHO consortium in which nearly all of the asthma birth cohorts utilized the ISAAC-WM. Our latent variable approach yields a continuous measure of wheezing illness severity that capitalizes on shared variance from the four wheezing-focused ISAAC-WM items. Our findings suggest that this latent variable approach results in stronger associations with established measures of wheezing illness severity than models using discrete severity variables based on the ISAAC-WM. Thus, for studies using the ISAAC-WM, the proposed latent variable approach may result in stronger tests of etiological theory than models using discrete severity variables.

It is important to note that the proposed SEM-based latent variable approach is intended for use in explanatory research models, where the goal is to test specific etiological hypotheses and theories. Latent variables do not yield unique scores for individual respondents [65]. Consequently, our proposed SEM-based latent variable model of wheezing severity could not be used in clinical practice to aid in diagnostic decisions or to make patient-specific predictions about risk for future adverse outcomes [65]. There are, however, a number of existing questionnaires measuring wheezing severity that were designed and validated for these purposes [18]. However, for researchers testing specific etiological models of wheezing illness development and/or outcomes with the ISAAC-WM, this approach could lead to stronger tests of theory. We provided programming code for both lavaan (open-source) and Mplus (proprietary) in online appendices to facilitate implementation in datasets utilizing the ISAAC-WM.

Despite its widespread use, this was the first study, to our knowledge, to test a model of wheezing illness severity using the ISAAC-WM. Our model was founded in the hypothesis that wheezing severity can be conceptualized as a continuous unidimensional latent variable and that the categorical ISAAC-WM items are surrogates, capturing measurable consequences of this unobserved variable. In each cohort, the χ^2^_GOF_ test, which is highly sensitive to model misspecifications with large sample sizes [66,67], found no significant evidence of discrepancies between the covariance matrixes implied by our proposed model of wheezing severity and the observed covariance matrixes.

This was also the first study to explicitly evaluate whether a model of pediatric wheezing severity was consistent over time (longitudinal invariance) and across child sexes. Our Cohort 1 analyses suggested that the factor structure (i.e., the relationship between the latent factor and its measured indicators) was stable over the first two years of life, allowing for meaningful evaluation of change in pediatric wheezing severity. Although males and females differed in their mean levels of wheezing severity, there was no evidence that the latent factor structure was inconsistent across child sexes. This means that a single model of wheezing severity in early childhood for girls and boys may be sufficient as long as analyses account for mean differences across sexes.

Finally, we demonstrated the benefits of using a continuous latent factor model within a structural equation modeling framework compared to a discrete wheezing severity variable in terms of its power to detect associations with future clinical outcomes. Going from the lowest to highest levels of the latent factor was associated with a greater increase in the estimated probability of future wheezing morbidity outcomes compared to going from the lowest to highest levels of a discrete severity variable. Thus, the latent factor approach may yield more powerful tests of etiological associations than categorical severity variables.

While there are a number of strengths of this study, including evaluation of the latent variable approach in three independent datasets, there are several limitations which should be considered. First, we relied exclusively on parent report for the measurement of both wheezing symptoms and validity outcomes; though the ISAAC-WM was designed and validated for this purpose. Second, we tested our factor model using just four ordinal indicators. More precise measurements of the indicators (e.g., exact counts of wheezing episodes) should result in better severity estimation. Greater precision might also be achieved by combining the ISAAC-WM items with other indicators of wheezing severity, assuming that they are theoretically and empirically commensurate with the proposed model. Third, our models were unidimensional and only captured variability in wheezing symptoms. Prior studies suggest that asthma is a multidimensional construct [16–18], with wheezing severity represented in one of the dimensions. Future studies should explore whether the proposed latent wheezing severity model can be combined with other symptom dimensions to provide a more comprehensive representation of asthma severity [68,69]. Fourth, the latent factor model does not discriminate as well among children who are low on the severity spectrum compared to those with moderate to severe symptoms. Adding indicators that better distinguish between children with mild symptoms would be desirable in future studies. Lastly, it is important to note that SEM generally requires large samples and may not be feasible in smaller studies [70].

## Conclusions

In conclusion, the proposed latent variable model of pediatric wheezing illness estimates wheezing severity as a continuous construct, is consistent with data from three independent cohorts, and is prospectively associated with asthma morbidity. This modeling approach can be applied in cohorts with ISAAC-WM data with a sufficient sample size by adapting the provided code. Using a latent severity approach provides more powerful testing of etiological hypotheses.

## Supporting information

S1 Fig Cohort 1: Associations between the latent wheezing severity factor and the ISAAC-WM indicators. The probability of being in each severity category on the ISAAC-WM severity items in year-one as a function of the latent wheezing illness factor. The probability of being in more severe categories increases with increasing levels of the latent factor for each ISAAC-WM item.
(TIFF)

S2 Fig Cohort 1 INSPIRE item information curves. This plot shows item information curves for each ISAAC-WM observed wheezing severity indicator at year one (panel a) and year-two (panel b). The reciprocal of the variance in the estimates (i.e., information) is plotted against levels of the latent factor, with higher levels of information indicating greater measurement precision. The latent factors are measured with greater precision at moderate to high levels of wheezing severity and are less good at distinguishing between individuals with low severity wheezing illness.
(TIFF)

S1 Appendix Model code and covariance matrixes for Cohort 1 analyses. (DOCX)

S2 Appendix Model code and covariance matrixes for Cohort 2 analyses. (DOCX)

S3 Appendix Model code and covariance matrixes for Cohort 3 analyses. (DOCX)

S1 File Data to reproduce Cohort 1 and Cohort 3 models. This zip file contains R data files with following information for all Cohort 1 and Cohort 3 models: observed covariance matrixes, thresholds, weighted least squares weight matrixes (the “wls.v” matrix in lavaan), sample asymptotic variance-covariance matrixes (the “gamma” matrix in lavaan), and sample sizes (n) associated with each model. As all models were covariance-structure models, these data should be sufficient to allow others to reproduce our results. Matrixes for each model are stored as lists within the R environment. The accompanying S1Code.R file provides R code for extracting summary statistics for each model.
(ZIP)
