# Supplementary figures and images for: A new model of wheezing severity in young children using the validated ISAAC wheezing module: A latent variable approach with validation in independent cohorts
Source: PLoS One. 2018 Apr 17;13(4):e0194739. doi: 10.1371/journal.pone.0194739 (PMC5903664; doi:10.1371/journal.pone.0194739)

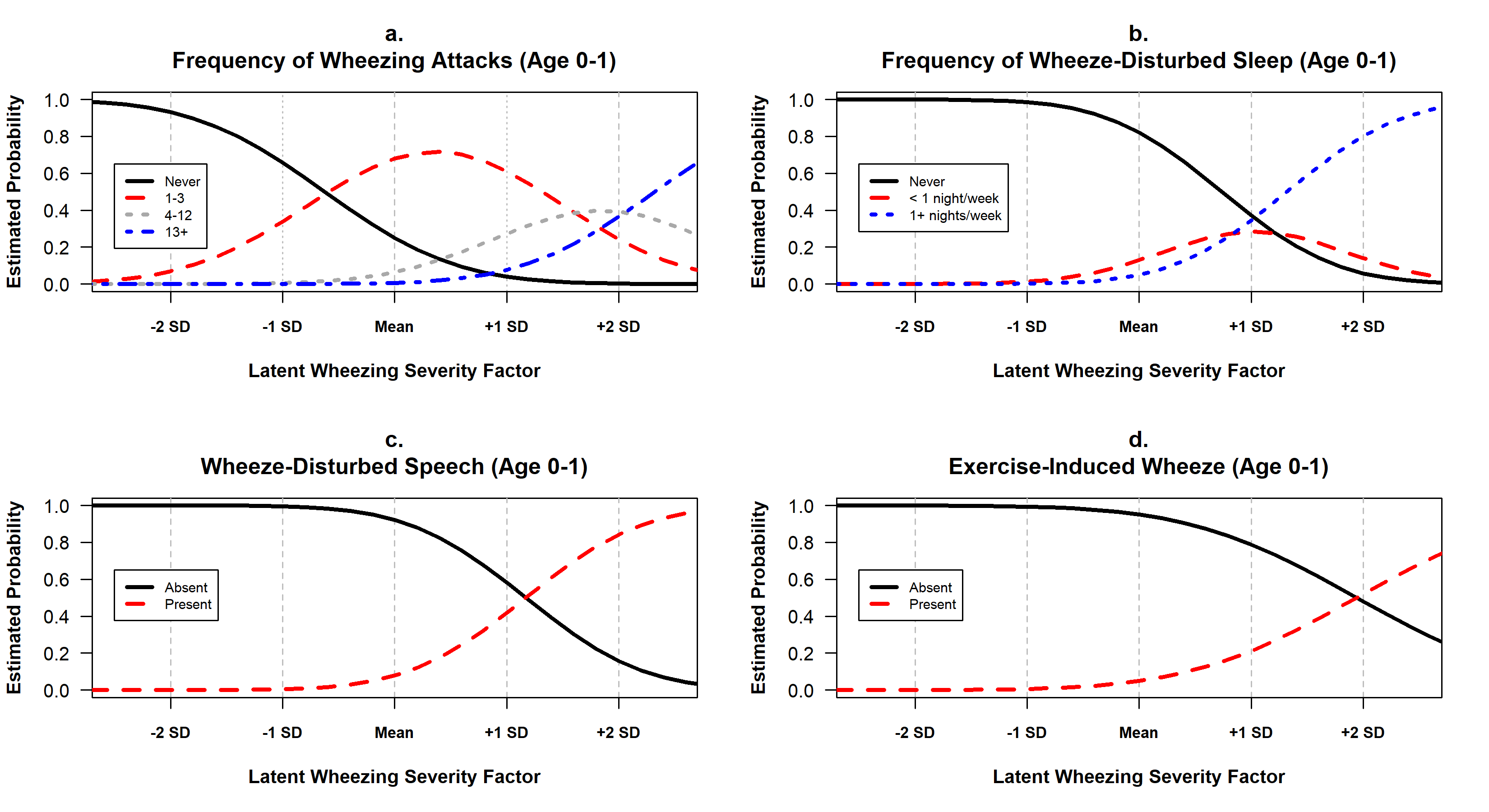

Supplement: S1 Fig — The probability of being in each severity category on the ISAAC-WM severity items in year-one as a function of the latent wheezing illness factor. The probability of being in more severe categories increases with increasing levels of the latent factor for each ISAAC-WM item. (TIFF) [file pone.0194739.s001.tiff]

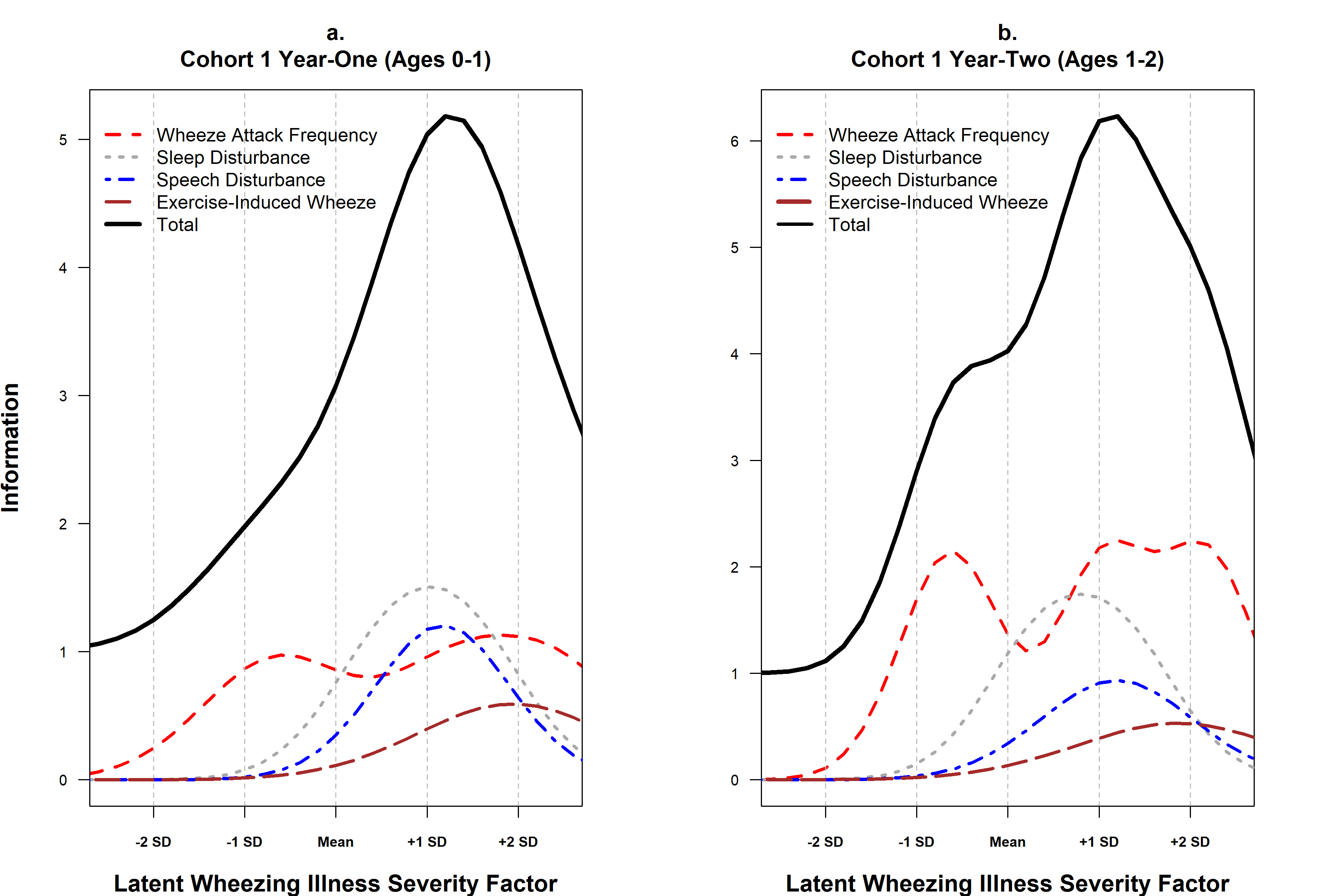

Supplement: S2 Fig — This plot shows item information curves for each ISAAC-WM observed wheezing severity indicator at year one (panel a) and year-two (panel b). The reciprocal of the variance in the estimates (i.e., information) is plotted against levels of the latent factor, with higher levels of information indicating greater measurement precision. The latent factors are measured with greater precision at moderate to high levels of wheezing severity and are less good at distinguishing between individuals with low severity wheezing illness. (TIFF) [file pone.0194739.s002.tiff]
