# Supplementary material for: A new model of wheezing severity in young children using the validated ISAAC wheezing module: A latent variable approach with validation in independent cohorts
Source: PLoS One. 2018 Apr 17;13(4):e0194739. doi: 10.1371/journal.pone.0194739 (PMC5903664; doi:10.1371/journal.pone.0194739)
Supplement: S1 Appendix — (DOCX) [file pone.0194739.s003.docx]

S1 Appendix: Cohort 1 Analyses

Table of Contents

[Contents 2](#_Toc502997841)

[Variable key 2](#_Toc502997842)

[Model 1.1 3](#_Toc502997843)

[m1.1: lavaan code 3](#_Toc502997844)

[m1.1: Sample statistics (observed covariance matrix and thresholds) 3](#_Toc502997845)

[m1.1: Model implied covariance matrix 3](#_Toc502997846)

[m1.1: Residual correlation matrix for model 1.1 4](#_Toc502997847)

[m1.1: Weight vector used in weighted least squares estimation for model 1.1 4](#_Toc502997848)

[Model 1.2 5](#_Toc502997849)

[m1.2: lavaan code 5](#_Toc502997850)

[m1.2: Sample statistics (observed covariance matrix and thresholds) 5](#_Toc502997851)

[m1.2: Model implied covariance matrix 5](#_Toc502997852)

[m1.2: Residual correlation matrix for model 1.2 5](#_Toc502997853)

[m1.2: Weight vector used in weighted least squares estimation for model 1.2 6](#_Toc502997854)

[Model 1.3 6](#_Toc502997855)

[m1.3: lavaan code 6](#_Toc502997856)

[m1.3: Sample statistics (observed covariance matrix and thresholds) 7](#_Toc502997857)

[m1.3: Model implied covariance matrix 7](#_Toc502997858)

[m1.3: Residual correlation matrix for model 1.3 8](#_Toc502997859)

[Model 1.4 8](#_Toc502997860)

[m1.4: lavaan code 8](#_Toc502997861)

[m1.4: Sample statistics (observed covariance matrix and thresholds) 8](#_Toc502997862)

[m1.4: Model implied covariance matrix 9](#_Toc502997863)

[m1.4: Residual correlation matrix for model 1.4 9](#_Toc502997864)

[Model 1.5 10](#_Toc502997865)

[m1.5: lavaan code 10](#_Toc502997866)

[m1.5: Sample statistics (observed covariance matrix and thresholds) 10](#_Toc502997867)

[m1.5: Model implied covariance matrix 11](#_Toc502997868)

[m1.5: Residual correlation matrix for model 1.5 11](#_Toc502997869)

[Model 1.6 11](#_Toc502997870)

[m1.6: lavaan code 11](#_Toc502997871)

[m1.6: Sample statistics (observed covariance matrix and thresholds) 11](#_Toc502997872)

[m1.6: Model implied covariance matrix 12](#_Toc502997873)

[m1.6: Residual correlation matrix for model 1.6 12](#_Toc502997874)

[Model 1.7 13](#_Toc502997875)

[m1.7: lavaan code 13](#_Toc502997876)

[m1.7: Sample statistics (observed covariance matrix and thresholds) 13](#_Toc502997877)

[m1.7: Model implied covariance matrix 14](#_Toc502997878)

[m1.7: Residual correlation matrix for model 1.7 14](#_Toc502997879)

[Model 1.8 14](#_Toc502997880)

[m1.8: lavaan code 14](#_Toc502997881)

[m1.8: Sample statistics (observed covariance matrix and thresholds) 15](#_Toc502997882)

[m1.8: Model implied covariance matrix 15](#_Toc502997883)

[m1.8: Residual correlation matrix for model 1.8 16](#_Toc502997884)

[Model 1.9 16](#_Toc502997885)

[m1.9: lavaan code 16](#_Toc502997886)

[m1.9: Sample statistics (observed covariance matrix and thresholds) 16](#_Toc502997887)

[m1.9: Model implied covariance matrix 17](#_Toc502997888)

[m1.9: Residual correlation matrix for model 1.9 17](#_Toc502997889)

# Contents

This file provides supplemental modeling information for the Cohort 1 analyses. We provide *lavaan* code for the primary Cohort 1 models (see Table 4 in the primary text). Additionally, we provide observed and model-implied covariance marixes for each model, as well as the residual correlation matrix. For models 1.1 and 1.2, we provide the weight matrix used in Weighted Least Squares estimation.

# Variable key

Below we define the observed variables used in the analyses. For detailed information about *lavaan* modeling code, see the [lavaan website](http://lavaan.ugent.be/).

- dnwhez12 = ISAAC-WM item from the one-year follow-up assessment asking about the frequency of wheezing attacks in the past year (4-level ordinal variable)
- dawake12 = ISAAC-WM item from the one-year follow-up assessment asking about the frequency of wheeze-induced sleep disturbance in the past year (3-level ordinal variable)
- dspeech12 = ISAAC-WM item from the one-year follow-up assessment asking about the frequency of wheeze-induced speech disturbance in the past year (2-level ordinal variable)
- dexwhez12 = ISAAC-WM item from the one-year follow-up assessment asking whether the child has experienced exercise-induced wheeze

# Model 1.1

## m1.1: lavaan code

m1.1syn <- '
whzsev12 =~ dnwhez12 + dawake12 + dspeech12 + dexwhez12
'

m1.1 <- cfa( m1.1syn, data = insp.isaac3, estimator = 'wlsmv', std.lv = T, missing = 'pairwise' )

## m1.1: Sample statistics (observed covariance matrix and thresholds)

## $cov
## dnwh12 dawk12 dspc12 dxwh12
## dnwhez12 1.000
## dawake12 0.590 1.000
## dspeech12 0.539 0.592 1.000
## dexwhez12 0.455 0.465 0.559 1.000
##
## $mean
## dnwhez12 dawake12 dspeech12 dexwhez12
## 0 0 0 0
##
## $th
## dnwhez12|t1 dnwhez12|t2 dnwhez12|t3 dawake12|t1 dawake12|t2
## -0.458 0.997 1.704 0.576 1.030
## dspeech12|t1 dexwhez12|t1
## 0.903 1.258

## m1.1: Model implied covariance matrix

## dnwh12 dawk12 dspc12 dxwh12
## dnwhez12 1.000
## dawake12 0.563 1.000
## dspeech12 0.576 0.602 1.000
## dexwhez12 0.475 0.497 0.509 1.000

## m1.1: Residual correlation matrix for model 1.1

## dnwh12 dawk12 dspc12 dxwh12
## dnwhez12 0.000
## dawake12 0.027 0.000
## dspeech12 -0.036 -0.009 0.000
## dexwhez12 -0.021 -0.032 0.051 0.000

## m1.1: Weight vector used in weighted least squares estimation for model 1.1

inspect( m1.1, 'wls.v' )

## [,1] [,2] [,3] [,4] [,5] [,6] [,7] [,8] [,9] [,10] [,11]
## [1,] 0.58 0.000 0.000 0.000 0.000 0.000 0.000 0.000 0.000 0.000 0.00
## [2,] 0.00 0.432 0.000 0.000 0.000 0.000 0.000 0.000 0.000 0.000 0.00
## [3,] 0.00 0.000 0.203 0.000 0.000 0.000 0.000 0.000 0.000 0.000 0.00
## [4,] 0.00 0.000 0.000 0.554 0.000 0.000 0.000 0.000 0.000 0.000 0.00
## [5,] 0.00 0.000 0.000 0.000 0.421 0.000 0.000 0.000 0.000 0.000 0.00
## [6,] 0.00 0.000 0.000 0.000 0.000 0.462 0.000 0.000 0.000 0.000 0.00
## [7,] 0.00 0.000 0.000 0.000 0.000 0.000 0.349 0.000 0.000 0.000 0.00
## [8,] 0.00 0.000 0.000 0.000 0.000 0.000 0.000 0.554 0.000 0.000 0.00
## [9,] 0.00 0.000 0.000 0.000 0.000 0.000 0.000 0.000 0.247 0.000 0.00
## [10,] 0.00 0.000 0.000 0.000 0.000 0.000 0.000 0.000 0.000 0.336 0.00
## [11,] 0.00 0.000 0.000 0.000 0.000 0.000 0.000 0.000 0.000 0.000 0.58
## [12,] 0.00 0.000 0.000 0.000 0.000 0.000 0.000 0.000 0.000 0.000 0.00
## [13,] 0.00 0.000 0.000 0.000 0.000 0.000 0.000 0.000 0.000 0.000 0.00
## [,12] [,13]
## [1,] 0.000 0.00
## [2,] 0.000 0.00
## [3,] 0.000 0.00
## [4,] 0.000 0.00
## [5,] 0.000 0.00
## [6,] 0.000 0.00
## [7,] 0.000 0.00
## [8,] 0.000 0.00
## [9,] 0.000 0.00
## [10,] 0.000 0.00
## [11,] 0.000 0.00
## [12,] 0.331 0.00
## [13,] 0.000 0.33

# Model 1.2

## m1.2: lavaan code

m1.2syn <- '
whzsev24 =~ dnwhez24 + dawake24 + dspeech24 + dexwhez24
'

m1.2 <- cfa( m1.2syn, data = insp.isaac3, estimator = 'wlsmv', std.lv = T, missing = 'pairwise' )

## m1.2: Sample statistics (observed covariance matrix and thresholds)

## $cov
## dnwh24 dawk24 dspc24 dxwh24
## dnwhez24 1.000
## dawake24 0.672 1.000
## dspeech24 0.599 0.624 1.000
## dexwhez24 0.594 0.471 0.387 1.000
##
## $mean
## dnwhez24 dawake24 dspeech24 dexwhez24
## 0 0 0 0
##
## $th
## dnwhez24|t1 dnwhez24|t2 dnwhez24|t3 dawake24|t1 dawake24|t2
## -0.529 0.924 1.845 0.365 0.916
## dspeech24|t1 dexwhez24|t1
## 0.872 1.166

## m1.2: Model implied covariance matrix

## dnwh24 dawk24 dspc24 dxwh24
## dnwhez24 1.000
## dawake24 0.690 1.000
## dspeech24 0.630 0.585 1.000
## dexwhez24 0.537 0.499 0.456 1.000

## m1.2: Residual correlation matrix for model 1.2

## dnwh24 dawk24 dspc24 dxwh24
## dnwhez24 0.000
## dawake24 -0.018 0.000
## dspeech24 -0.031 0.039 0.000
## dexwhez24 0.057 -0.028 -0.069 0.000

## m1.2: Weight vector used in weighted least squares estimation for model 1.2

inspect( m1.2, 'wls.v' )

## [,1] [,2] [,3] [,4] [,5] [,6] [,7] [,8] [,9] [,10] [,11]
## [1,] 0.48 0.000 0.00 0.000 0.000 0.000 0.000 0.000 0.000 0.00 0.00
## [2,] 0.00 0.387 0.00 0.000 0.000 0.000 0.000 0.000 0.000 0.00 0.00
## [3,] 0.00 0.000 0.14 0.000 0.000 0.000 0.000 0.000 0.000 0.00 0.00
## [4,] 0.00 0.000 0.00 0.507 0.000 0.000 0.000 0.000 0.000 0.00 0.00
## [5,] 0.00 0.000 0.00 0.000 0.389 0.000 0.000 0.000 0.000 0.00 0.00
## [6,] 0.00 0.000 0.00 0.000 0.000 0.401 0.000 0.000 0.000 0.00 0.00
## [7,] 0.00 0.000 0.00 0.000 0.000 0.000 0.381 0.000 0.000 0.00 0.00
## [8,] 0.00 0.000 0.00 0.000 0.000 0.000 0.000 0.614 0.000 0.00 0.00
## [9,] 0.00 0.000 0.00 0.000 0.000 0.000 0.000 0.000 0.253 0.00 0.00
## [10,] 0.00 0.000 0.00 0.000 0.000 0.000 0.000 0.000 0.000 0.41 0.00
## [11,] 0.00 0.000 0.00 0.000 0.000 0.000 0.000 0.000 0.000 0.00 0.55
## [12,] 0.00 0.000 0.00 0.000 0.000 0.000 0.000 0.000 0.000 0.00 0.00
## [13,] 0.00 0.000 0.00 0.000 0.000 0.000 0.000 0.000 0.000 0.00 0.00
## [,12] [,13]
## [1,] 0.000 0.000
## [2,] 0.000 0.000
## [3,] 0.000 0.000
## [4,] 0.000 0.000
## [5,] 0.000 0.000
## [6,] 0.000 0.000
## [7,] 0.000 0.000
## [8,] 0.000 0.000
## [9,] 0.000 0.000
## [10,] 0.000 0.000
## [11,] 0.000 0.000
## [12,] 0.351 0.000
## [13,] 0.000 0.243

# Model 1.3

## m1.3: lavaan code

m1.3syn <- '
whzsev12 =~ dnwhez12 + dawake12 + dspeech12 + dexwhez12
whzsev12 ~ masthma + msmokepreg + medicaid + cesarean + male + married + cblack + cother
resphosp12 ~ whzsev12 + masthma + msmokepreg + medicaid + cesarean + male +
 married + cblack + cother
'

m1.3 <- cfa( m1.3syn, data = insp.isaac3, estimator = 'wlsmv', std.lv = T, missing = 'pairwise' )

## m1.3: Sample statistics (observed covariance matrix and thresholds)

## $res.cov
## dnwh12 dawk12 dspc12 dxwh12 rsph12
## dnwhez12 1.000
## dawake12 0.590 1.000
## dspeech12 0.541 0.595 1.000
## dexwhez12 0.460 0.473 0.566 1.000
## resphosp12 0.300 0.299 0.481 0.422 1.000
##
## $res.int
## dnwhez12 dawake12 dspeech12 dexwhez12 resphosp12
## 0 0 0 0 0
##
## $res.th
## dnwhez12|t1 dnwhez12|t2 dnwhez12|t3 dawake12|t1 dawake12|t2
## -0.317 1.162 1.882 0.705 1.164
## dspeech12|t1 dexwhez12|t1 resphosp12|t1
## 0.551 1.243 1.147
##
## $res.slopes
## masthm msmkpr medicd cesarn male marrid cblack cother
## dnwhez12 0.225 0.110 0.118 0.139 0.113 -0.117 -0.128 -0.163
## dawake12 0.017 0.202 0.156 0.015 -0.003 0.035 -0.070 -0.174
## dspeech12 0.062 0.174 -0.177 -0.092 -0.067 -0.306 -0.155 -0.439
## dexwhez12 0.030 0.028 -0.063 0.037 -0.006 -0.123 0.285 -0.302
## resphosp12 -0.081 0.299 0.093 -0.102 -0.098 -0.016 0.377 -0.157
##
## $cov.x
## masthm msmkpr medicd cesarn male marrid cblack cother
## masthma 0.180
## msmokepreg 0.007 0.167
## medicaid 0.001 0.052 0.245
## cesarean 0.007 -0.001 -0.024 0.225
## male 0.005 -0.006 -0.005 0.000 0.236
## married -0.008 -0.058 -0.142 0.011 0.002 0.247
## cblack 0.012 -0.001 0.067 -0.004 -0.001 -0.086 0.190
## cother 0.004 -0.005 0.010 -0.006 -0.003 -0.012 -0.002 0.074

## m1.3: Model implied covariance matrix

## dnwh12 dawk12 dspc12 dxwh12 rsph12
## dnwhez12 1.000
## dawake12 0.497 1.000
## dspeech12 0.696 0.584 1.000
## dexwhez12 0.507 0.425 0.595 1.000
## resphosp12 0.391 0.328 0.459 0.334 1.000

## m1.3: Residual correlation matrix for model 1.3

## dnwh12 dawk12 dspc12 dxwh12 rsph12
## dnwhez12 0.000
## dawake12 0.093 0.000
## dspeech12 -0.155 0.011 0.000
## dexwhez12 -0.047 0.047 -0.030 0.000
## resphosp12 -0.090 -0.029 0.022 0.088 0.000

# Model 1.4

## m1.4: lavaan code

m1.4syn <- '
whzsev24 =~ dnwhez24 + dawake24 + dspeech24 + dexwhez24
whzsev24 ~ masthma + msmokepreg + medicaid + cesarean + male + married + cblack + cother
resphosp24 ~ whzsev24 + masthma + msmokepreg + medicaid + cesarean + male + married + cblack + cother
'

m1.4 <- cfa( m1.4syn, data = insp.isaac3, estimator = 'wlsmv', std.lv = T, missing = 'pairwise' )

## m1.4: Sample statistics (observed covariance matrix and thresholds)

## $res.cov
## dnwh24 dawk24 dspc24 dxwh24 rsph24
## dnwhez24 1.000
## dawake24 0.664 1.000
## dspeech24 0.610 0.631 1.000
## dexwhez24 0.576 0.457 0.437 1.000
## resphosp24 0.271 0.254 0.539 0.344 1.000
##
## $res.int
## dnwhez24 dawake24 dspeech24 dexwhez24 resphosp24
## 0 0 0 0 0
##
## $res.th
## dnwhez24|t1 dnwhez24|t2 dnwhez24|t3 dawake24|t1 dawake24|t2
## -0.357 1.128 2.083 0.577 1.139
## dspeech24|t1 dexwhez24|t1 resphosp24|t1
## 1.006 1.470 1.753
##
## $res.slopes
## masthm msmkpr medicd cesarn male marrid cblack cother
## dnwhez24 0.402 0.058 -0.004 0.102 0.145 -0.094 0.063 -0.155
## dawake24 0.276 -0.175 0.114 -0.015 0.169 -0.052 0.147 -0.001
## dspeech24 0.373 -0.241 -0.044 0.000 0.374 -0.134 -0.252 -0.185
## dexwhez24 0.230 0.016 0.102 0.149 0.063 -0.187 0.506 0.185
## resphosp24 -0.106 -0.020 0.390 0.141 -0.084 0.081 0.150 -0.600
##
## $cov.x
## masthm msmkpr medicd cesarn male marrid cblack cother
## masthma 0.180
## msmokepreg 0.007 0.167
## medicaid 0.001 0.052 0.245
## cesarean 0.007 -0.001 -0.024 0.225
## male 0.005 -0.006 -0.005 0.000 0.236
## married -0.008 -0.058 -0.142 0.011 0.002 0.247
## cblack 0.012 -0.001 0.067 -0.004 -0.001 -0.086 0.190
## cother 0.004 -0.005 0.010 -0.006 -0.003 -0.012 -0.002 0.074

## m1.4: Model implied covariance matrix

## dnwh24 dawk24 dspc24 dxwh24 rsph24
## dnwhez24 1.000
## dawake24 0.541 1.000
## dspeech24 0.738 0.621 1.000
## dexwhez24 0.550 0.463 0.632 1.000
## resphosp24 0.363 0.306 0.418 0.311 1.000

## m1.4: Residual correlation matrix for model 1.4

## dnwh24 dawk24 dspc24 dxwh24 rsph24
## dnwhez24 0.000
## dawake24 0.123 0.000
## dspeech24 -0.128 0.010 0.000
## dexwhez24 0.026 -0.007 -0.195 0.000
## resphosp24 -0.093 -0.052 0.122 0.033 0.000

# Model 1.5

## m1.5: lavaan code

m1.5syn <- '
whzsev12 =~ dnwhez12 + dawake12 + dspeech12 + dexwhez12
whzsev12 ~ masthma + msmokepreg + medicaid + cesarean + male + married + cblack + cother
whzmed12 ~ whzsev12 + masthma + msmokepreg + medicaid + cesarean + male + married + cblack + cother
'

m1.5 <- cfa( m1.5syn, data = insp.isaac3, estimator = 'wlsmv', std.lv = T, missing = 'pairwise' )

## m1.5: Sample statistics (observed covariance matrix and thresholds)

## $res.cov
## dnwh12 dawk12 dspc12 dxwh12 whzm12
## dnwhez12 1.000
## dawake12 0.590 1.000
## dspeech12 0.541 0.595 1.000
## dexwhez12 0.460 0.473 0.566 1.000
## whzmed12 0.584 0.452 0.506 0.447 1.000
##
## $res.int
## dnwhez12 dawake12 dspeech12 dexwhez12 whzmed12
## 0 0 0 0 0
##
## $res.th
## dnwhez12|t1 dnwhez12|t2 dnwhez12|t3 dawake12|t1 dawake12|t2
## -0.317 1.162 1.882 0.705 1.164
## dspeech12|t1 dexwhez12|t1 whzmed12|t1
## 0.551 1.243 0.616
##
## $res.slopes
## masthm msmkpr medicd cesarn male marrid cblack cother
## dnwhez12 0.225 0.110 0.118 0.139 0.113 -0.117 -0.128 -0.163
## dawake12 0.017 0.202 0.156 0.015 -0.003 0.035 -0.070 -0.174
## dspeech12 0.062 0.174 -0.177 -0.092 -0.067 -0.306 -0.155 -0.439
## dexwhez12 0.030 0.028 -0.063 0.037 -0.006 -0.123 0.285 -0.302
## whzmed12 0.212 0.104 0.048 0.100 0.167 -0.142 -0.261 -0.025
##
## $cov.x
## masthm msmkpr medicd cesarn male marrid cblack cother
## masthma 0.180
## msmokepreg 0.007 0.167
## medicaid 0.001 0.052 0.245
## cesarean 0.007 -0.001 -0.024 0.225
## male 0.005 -0.006 -0.005 0.000 0.236
## married -0.008 -0.058 -0.142 0.011 0.002 0.247
## cblack 0.012 -0.001 0.067 -0.004 -0.001 -0.086 0.190
## cother 0.004 -0.005 0.010 -0.006 -0.003 -0.012 -0.002 0.074

## m1.5: Model implied covariance matrix

## dnwh12 dawk12 dspc12 dxwh12 whzm12
## dnwhez12 1.000
## dawake12 0.600 1.000
## dspeech12 0.727 0.575 1.000
## dexwhez12 0.542 0.428 0.519 1.000
## whzmed12 0.576 0.455 0.551 0.411 1.000

## m1.5: Residual correlation matrix for model 1.5

## dnwh12 dawk12 dspc12 dxwh12 whzm12
## dnwhez12 0.000
## dawake12 -0.010 0.000
## dspeech12 -0.186 0.020 0.000
## dexwhez12 -0.081 0.045 0.047 0.000
## whzmed12 0.008 -0.003 -0.046 0.036 0.000

# Model 1.6

## m1.6: lavaan code

m1.6syn <- '
whzsev24 =~ dnwhez24 + dawake24 + dspeech24 + dexwhez24
whzsev24 ~ masthma + msmokepreg + medicaid + cesarean + male + married + cblack + cother
whzmed24 ~ whzsev24 + masthma + msmokepreg + medicaid + cesarean + male + married + cblack + cother
'

m1.6 <- cfa( m1.6syn, data = insp.isaac3, estimator = 'wlsmv', std.lv = T, missing = 'pairwise' )

## m1.6: Sample statistics (observed covariance matrix and thresholds)

## $res.cov
## dnwh24 dawk24 dspc24 dxwh24 whzm24
## dnwhez24 1.000
## dawake24 0.664 1.000
## dspeech24 0.610 0.631 1.000
## dexwhez24 0.576 0.457 0.437 1.000
## whzmed24 0.672 0.604 0.602 0.426 1.000
##
## $res.int
## dnwhez24 dawake24 dspeech24 dexwhez24 whzmed24
## 0 0 0 0 0
##
## $res.th
## dnwhez24|t1 dnwhez24|t2 dnwhez24|t3 dawake24|t1 dawake24|t2
## -0.357 1.128 2.083 0.577 1.139
## dspeech24|t1 dexwhez24|t1 whzmed24|t1
## 1.006 1.470 0.087
##
## $res.slopes
## masthm msmkpr medicd cesarn male marrid cblack cother
## dnwhez24 0.402 0.058 -0.004 0.102 0.145 -0.094 0.063 -0.155
## dawake24 0.276 -0.175 0.114 -0.015 0.169 -0.052 0.147 -0.001
## dspeech24 0.373 -0.241 -0.044 0.000 0.374 -0.134 -0.252 -0.185
## dexwhez24 0.230 0.016 0.102 0.149 0.063 -0.187 0.506 0.185
## whzmed24 0.107 0.109 -0.157 0.141 0.251 -0.124 -0.102 -0.167
##
## $cov.x
## masthm msmkpr medicd cesarn male marrid cblack cother
## masthma 0.180
## msmokepreg 0.007 0.167
## medicaid 0.001 0.052 0.245
## cesarean 0.007 -0.001 -0.024 0.225
## male 0.005 -0.006 -0.005 0.000 0.236
## married -0.008 -0.058 -0.142 0.011 0.002 0.247
## cblack 0.012 -0.001 0.067 -0.004 -0.001 -0.086 0.190
## cother 0.004 -0.005 0.010 -0.006 -0.003 -0.012 -0.002 0.074

## m1.6: Model implied covariance matrix

## dnwh24 dawk24 dspc24 dxwh24 whzm24
## dnwhez24 1.000
## dawake24 0.671 1.000
## dspeech24 0.708 0.622 1.000
## dexwhez24 0.556 0.488 0.515 1.000
## whzmed24 0.671 0.589 0.621 0.488 1.000

## m1.6: Residual correlation matrix for model 1.6

## dnwh24 dawk24 dspc24 dxwh24 whzm24
## dnwhez24 0.000
## dawake24 -0.007 0.000
## dspeech24 -0.098 0.009 0.000
## dexwhez24 0.020 -0.032 -0.078 0.000
## whzmed24 0.001 0.015 -0.020 -0.062 0.000

# Model 1.7

## m1.7: lavaan code

m1.7syn <- '
whzsev24 =~ dnwhez24 + dawake24 + dspeech24 + dexwhez24
whzsev24 ~ masthma + msmokepreg + medicaid + cesarean + male + married + cblack + cother
y3asthdx ~ whzsev24 + masthma + msmokepreg + medicaid + cesarean + male + married + cblack + cother
'

m1.7 <- cfa(m1.7syn, data = insp.isaac3, estimator = 'wlsmv', ordered = 'y3asthdx', missing = 'pairwise' )

## m1.7: Sample statistics (observed covariance matrix and thresholds)

## $res.cov
## dnwh24 dawk24 dspc24 dxwh24 y3sthd
## dnwhez24 1.000
## dawake24 0.664 1.000
## dspeech24 0.610 0.631 1.000
## dexwhez24 0.576 0.457 0.437 1.000
## y3asthdx 0.432 0.503 0.420 0.327 1.000
##
## $res.int
## dnwhez24 dawake24 dspeech24 dexwhez24 y3asthdx
## 0 0 0 0 0
##
## $res.th
## dnwhez24|t1 dnwhez24|t2 dnwhez24|t3 dawake24|t1 dawake24|t2
## -0.357 1.128 2.083 0.577 1.139
## dspeech24|t1 dexwhez24|t1 y3asthdx|t1
## 1.006 1.470 1.595
##
## $res.slopes
## masthm msmkpr medicd cesarn male marrid cblack cother
## dnwhez24 0.402 0.058 -0.004 0.102 0.145 -0.094 0.063 -0.155
## dawake24 0.276 -0.175 0.114 -0.015 0.169 -0.052 0.147 -0.001
## dspeech24 0.373 -0.241 -0.044 0.000 0.374 -0.134 -0.252 -0.185
## dexwhez24 0.230 0.016 0.102 0.149 0.063 -0.187 0.506 0.185
## y3asthdx 0.425 0.007 0.121 -0.079 0.011 -0.054 0.615 0.118
##
## $cov.x
## masthm msmkpr medicd cesarn male marrid cblack cother
## masthma 0.180
## msmokepreg 0.007 0.167
## medicaid 0.001 0.052 0.245
## cesarean 0.007 -0.001 -0.024 0.225
## male 0.005 -0.006 -0.005 0.000 0.236
## married -0.008 -0.058 -0.142 0.011 0.002 0.247
## cblack 0.012 -0.001 0.067 -0.004 -0.001 -0.086 0.190
## cother 0.004 -0.005 0.010 -0.006 -0.003 -0.012 -0.002 0.074

## m1.7: Model implied covariance matrix

## dnwh24 dawk24 dspc24 dxwh24 y3sthd
## dnwhez24 1.000
## dawake24 0.650 1.000
## dspeech24 0.712 0.620 1.000
## dexwhez24 0.557 0.485 0.531 1.000
## y3asthdx 0.458 0.399 0.437 0.342 1.000

## m1.7: Residual correlation matrix for model 1.7

## dnwh24 dawk24 dspc24 dxwh24 y3sthd
## dnwhez24 0.000
## dawake24 0.014 0.000
## dspeech24 -0.102 0.011 0.000
## dexwhez24 0.019 -0.029 -0.094 0.000
## y3asthdx -0.026 0.105 -0.017 -0.015 0.000

# Model 1.8

## m1.8: lavaan code

m1.8syn <- '
whzsev24 =~ dnwhez24 + dawake24 + dspeech24 + dexwhez24
whzsev24 ~ masthma + msmokepreg + medicaid + cesarean + male + married + cblack + cother
y3steroidso ~ whzsev24 + masthma + msmokepreg + medicaid + cesarean + male + married + cblack + cother
'

m1.8 <- cfa(m1.8syn, data = insp.isaac3, estimator = 'wlsmv', ordered = 'y3steroidso', missing = 'pairwise' )

## m1.8: Sample statistics (observed covariance matrix and thresholds)

## $res.cov
## dnwh24 dawk24 dspc24 dxwh24 y3strd
## dnwhez24 1.000
## dawake24 0.664 1.000
## dspeech24 0.610 0.631 1.000
## dexwhez24 0.576 0.457 0.437 1.000
## y3steroidso 0.466 0.417 0.351 0.363 1.000
##
## $res.int
## dnwhez24 dawake24 dspeech24 dexwhez24 y3steroidso
## 0 0 0 0 0
##
## $res.th
## dnwhez24|t1 dnwhez24|t2 dnwhez24|t3 dawake24|t1 dawake24|t2
## -0.357 1.128 2.083 0.577 1.139
## dspeech24|t1 dexwhez24|t1 y3steroidso|t1 y3steroidso|t2
## 1.006 1.470 0.534 1.654
##
## $res.slopes
## masthm msmkpr medicd cesarn male marrid cblack cother
## dnwhez24 0.402 0.058 -0.004 0.102 0.145 -0.094 0.063 -0.155
## dawake24 0.276 -0.175 0.114 -0.015 0.169 -0.052 0.147 -0.001
## dspeech24 0.373 -0.241 -0.044 0.000 0.374 -0.134 -0.252 -0.185
## dexwhez24 0.230 0.016 0.102 0.149 0.063 -0.187 0.506 0.185
## y3steroidso 0.454 0.083 -0.215 -0.094 -0.017 -0.153 0.073 -0.130
##
## $cov.x
## masthm msmkpr medicd cesarn male marrid cblack cother
## masthma 0.180
## msmokepreg 0.007 0.167
## medicaid 0.001 0.052 0.245
## cesarean 0.007 -0.001 -0.024 0.225
## male 0.005 -0.006 -0.005 0.000 0.236
## married -0.008 -0.058 -0.142 0.011 0.002 0.247
## cblack 0.012 -0.001 0.067 -0.004 -0.001 -0.086 0.190
## cother 0.004 -0.005 0.010 -0.006 -0.003 -0.012 -0.002 0.074

## m1.8: Model implied covariance matrix

## dnwh24 dawk24 dspc24 dxwh24 y3strd
## dnwhez24 1.000
## dawake24 0.636 1.000
## dspeech24 0.702 0.605 1.000
## dexwhez24 0.564 0.486 0.537 1.000
## y3steroidso 0.454 0.392 0.433 0.347 1.000

## m1.8: Residual correlation matrix for model 1.8

## dnwh24 dawk24 dspc24 dxwh24 y3strd
## dnwhez24 0.000
## dawake24 0.028 0.000
## dspeech24 -0.092 0.026 0.000
## dexwhez24 0.012 -0.029 -0.099 0.000
## y3steroidso 0.011 0.025 -0.081 0.015 0.000

# Model 1.9

## m1.9: lavaan code

m1.9syn <- '
whzsev24 =~ dnwhez24 + dawake24 + dspeech24 + dexwhez24
whzsev24 ~ masthma + msmokepreg + medicaid + cesarean + male + married + cblack + cother
y3whzvisito ~ whzsev24 + masthma + msmokepreg + medicaid + cesarean + male + married + cblack + cother
'

m1.9 <- cfa(m1.9syn, data = insp.isaac3, estimator = 'wlsmv', ordered = 'y3whzvisito', missing = 'pairwise' )

## m1.9: Sample statistics (observed covariance matrix and thresholds)

## $res.cov
## dnwh24 dawk24 dspc24 dxwh24 y3whzv
## dnwhez24 1.000
## dawake24 0.664 1.000
## dspeech24 0.610 0.631 1.000
## dexwhez24 0.576 0.457 0.437 1.000
## y3whzvisito 0.461 0.423 0.397 0.331 1.000
##
## $res.int
## dnwhez24 dawake24 dspeech24 dexwhez24 y3whzvisito
## 0 0 0 0 0
##
## $res.th
## dnwhez24|t1 dnwhez24|t2 dnwhez24|t3 dawake24|t1 dawake24|t2
## -0.357 1.128 2.083 0.577 1.139
## dspeech24|t1 dexwhez24|t1 y3whzvisito|t1 y3whzvisito|t2
## 1.006 1.470 0.447 1.610
##
## $res.slopes
## masthm msmkpr medicd cesarn male marrid cblack cother
## dnwhez24 0.402 0.058 -0.004 0.102 0.145 -0.094 0.063 -0.155
## dawake24 0.276 -0.175 0.114 -0.015 0.169 -0.052 0.147 -0.001
## dspeech24 0.373 -0.241 -0.044 0.000 0.374 -0.134 -0.252 -0.185
## dexwhez24 0.230 0.016 0.102 0.149 0.063 -0.187 0.506 0.185
## y3whzvisito 0.375 -0.010 -0.223 -0.195 0.115 -0.345 0.231 -0.165
##
## $cov.x
## masthm msmkpr medicd cesarn male marrid cblack cother
## masthma 0.180
## msmokepreg 0.007 0.167
## medicaid 0.001 0.052 0.245
## cesarean 0.007 -0.001 -0.024 0.225
## male 0.005 -0.006 -0.005 0.000 0.236
## married -0.008 -0.058 -0.142 0.011 0.002 0.247
## cblack 0.012 -0.001 0.067 -0.004 -0.001 -0.086 0.190
## cother 0.004 -0.005 0.010 -0.006 -0.003 -0.012 -0.002 0.074

## m1.9: Model implied covariance matrix

## dnwh24 dawk24 dspc24 dxwh24 y3whzv
## dnwhez24 1.000
## dawake24 0.650 1.000
## dspeech24 0.703 0.608 1.000
## dexwhez24 0.562 0.487 0.526 1.000
## y3whzvisito 0.459 0.397 0.430 0.344 1.000

## m1.9: Residual correlation matrix for model 1.9

## dnwh24 dawk24 dspc24 dxwh24 y3whzv
## dnwhez24 0.000
## dawake24 0.014 0.000
## dspeech24 -0.093 0.023 0.000
## dexwhez24 0.014 -0.030 -0.089 0.000
## y3whzvisito 0.003 0.026 -0.032 -0.013 0.000
