# Supplementary material for: A new model of wheezing severity in young children using the validated ISAAC wheezing module: A latent variable approach with validation in independent cohorts
Source: PLoS One. 2018 Apr 17;13(4):e0194739. doi: 10.1371/journal.pone.0194739 (PMC5903664; doi:10.1371/journal.pone.0194739)
Supplement: S2 Appendix — (DOCX) [file pone.0194739.s004.docx]

S2 Appendix: Cohort 2 Analyses

| **Contents** | |
| --- | --- |
| ISAAC Phase I (Cohort 2) data preparation in R……………………………………… | 2-3 |
| Mplus code for Model 2.1………………………………………………………………... | 4 |
| Mplus code for Model 2.2………………………………………………………………... | 5 |

| **ISAAC Phase I (Cohort 2) data preparation in R** |
| --- |
| ISAAC Phase I data were publicly available and downloaded on November 7, 2016 from: <http://isaac.auckland.ac.nz/phases/phaseone/results/resultsIndv.php>. The Coding and Data Transfer Manual is available at: <http://isaac.auckland.ac.nz/phases/phaseone/phaseone.html>.  **R Code for Data Preparation**  ##############################  # Read in and merge data sets  ##############################  ## Load ISAAC Phase 1 data sets for 6-7 year-old N. American children into R  barb67 <- read.csv( 'barb67.csv', header = T )  ham67 <- read.csv( 'ham67.csv', header = T )  sask67 <- read.csv( 'sask67.csv', header = T )  ## Merge the data sets together (by rows) to create a single data frame  isaac <- rbind( barb67, ham67, sask67 )  ##############################  # Recode relevant variables  ##############################  ## Make sure there is no overlap in school numbers across countries  ## by adding 1000 to all number within school 173  isaac$dschool <- isaac$school  isaac$dschool[ isaac$country == 173 ] <- isaac$school[ isaac$country == 173 ] + 1000  ## Child sex: make female = 0 and male = 1  isaac$dsex <- ifelse( isaac$sex == 9, NA, 2 - isaac$sex )  with(isaac, table( sex, dsex ) )  ## ISAAC-WM item 1: Has your child ever wheezed?  # Recode: missing = NA; No = 0; 1 = Yes  table( isaac$dwhezev <- ifelse( isaac$whezev == 9, NA, 2 - isaac$whezev ) )  ## ISAAC-WM item 2: Has your child wheezed in past 12 months?  # Recode: missing = NA; No = 0; 1 = Yes  table( isaac$dwhez12 <- ifelse( isaac$whez12 == 9, NA, 2 - isaac$whez12 ) )  # If parent denied ever wheezing (dwhezev==0) then code 0 for this item  table( isaac$dwhez12 <- ifelse( isaac$dwhezev == 0, 0, isaac$dwhez12 ) )  ## ISAAC-WM item 3: How many attacks of wheezing in past 12 months?  # Recode: missing = NA; None = 0; 1-3 = 1; 4-12 = 2; 13+ = 3  table( isaac$dnwhez12 <- ifelse( isaac$nwhez12 == 9, NA, isaac$nwhez12 - 1 ) )  # If denied ever wheezing (dwhezev==0) or wheezing in past 12 months (dwhez12==0) then code 0  table( isaac$dnwhez12 <- ifelse( isaac$dwhezev == 0 \| isaac$dwhez12 == 0, 0, isaac$dnwhez12 ) )  ## ISAAC-WM item 4: Sleep disturbed due to wheezing in past 12 months?  # Recode: missing = NA; Never = 0; < 1 night/week = 1; 1+ nights/week = 2  table( isaac$dawake12 <- ifelse( isaac$awake12 == 9, NA, isaac$awake12 - 1 ) )  # If denied ever wheezing (dwhezev==0) or wheezing in past 12 months (dwhez12==0) then code 0  table( isaac$dawake12 <- ifelse( isaac$dwhezev == 0 \| isaac$dwhez12 == 0, 0, isaac$dawake12 ) )  ## ISAAC-WM item 5: Speech ever limited due to wheezing in past 12 months?  # Recode: missing = NA; No = 0; Yes = 1  table( isaac$dspeech12 <- ifelse( isaac$speech12 == 9, NA, 2 - isaac$speech12 ) )  # If denied ever wheezing (dwhezev==0) or wheezing in past 12 months (dwhez12==0) then code 0  table( isaac$dspeech12 <- ifelse( isaac$dwhezev == 0 \| isaac$dwhez12 == 0, 0, isaac$dspeech12 ) )  ## ISAAC-WM item 7: Exercise induced wheeze?  # Recode: missing = NA; No = 0; Yes = 1  table( isaac$dexwhez12 <- ifelse( isaac$exwhez12 == 9, NA, 2 - isaac$exwhez12 ) )  with(isaac, table( exwhez12, dexwhez12 ) )  summary( isaac$dexwhez12 )  ## Recode study centre variable as three dummy variables with values 0 and 1  table( isaac$c1 <- ifelse( isaac$centre == 1, 1, 0 ) )  table( isaac$c2 <- ifelse( isaac$centre == 2, 1, 0 ) )  table( isaac$c99 <- ifelse( isaac$centre == 99, 1, 0 ) )  #########################################################################  # Create new data frame (isaac1) with only children who have ever wheezed  #########################################################################  isaac1 <- subset( isaac, dwhezev == 1 )  ################################################################################  # Create Mplus data and input files using the MplusAutomation R package (Hallquist & Wiley, 2017)  ################################################################################  Install.packages( ‘MplusAutomation’ )  library( MplusAutomation )  prepareMplusData( isaac1, file = 'isaac1.dat' ) |

| **Mplus code for Model 2.1:** Cohort 2 (ISAAC Phase 1) multilevel SEM model of wheezing severity (see [www.statmodel.com](http://www.statmodel.com) for coding details; see Code E1 for variable key) |
| --- |
| TITLE: m2.1 -- saturated between-schools model;  DATA: FILE = "isaac1.dat";  VARIABLE:  NAMES = form version country centre agegrp school serial dint age agemonths sex  whezev whez12 nwhez12 awake12 speech12 asthmaev exwhez12 cough12 pnoseev  pnose12 ieyes12 pnosejan pnosefeb pnosemar pnoseapr pnosemay pnosejun pnosejul  pnoseaug pnosesep pnoseoct pnosenov pnosedec iactiv12 hfeverev rashev rash12  sitesev rashage rclear12 rawake12 eczemaev brthev brth12 brth1m exbrthev  exbrth12 exbrth1m wwokenev wwoken12 wwoken1m cwokenev cwoken12 cwoken1m  sabrthev sabrth12 sabrth1m dschool dsex dwhezev dwhez12 dnwhez12 dawake12  dspeech12 dexwhez12 c1 c2 c99;  MISSING=.;  CLUSTER = dschool;  USEVAR=dnwhez12 dawake12 dspeech12 dexwhez12 c2 c99;  BETWEEN = c2 c99;  CATEGORICAL = dnwhez12 dawake12 dspeech12 dexwhez12;  ANALYSIS: type = twolevel; estimator = wlsmv;  MODEL:  %WITHIN%  whzw BY dnwhez12* dawake12* dspeech12* dexwhez12*;  whzw @1;  %BETWEEN%  dnwhez12 WITH dawake12 dspeech12 dexwhez12;  dawake12 WITH dspeech12 dexwhez12;  dspeech12 WITH dexwhez12;  dnwhez12 dawake12 dspeech12 dexwhez12 ON c2 c99;  OUTPUT: sampstat cinterval residual stand(stdy);  PLOT: type = plot1 plot2 plot3; |

| **Mplus code for Model 2.2:** Cohort 2 (ISAAC Phase 1) multilevel SEM model regression wheezing severity on child sex on the within-schools level of analysis. |
| --- |
| TITLE: m2.1 -- saturated between-schools model;  DATA: FILE = "isaac1.dat";  VARIABLE:  NAMES = form version country centre agegrp school serial dint age agemonths sex  whezev whez12 nwhez12 awake12 speech12 asthmaev exwhez12 cough12 pnoseev  pnose12 ieyes12 pnosejan pnosefeb pnosemar pnoseapr pnosemay pnosejun pnosejul  pnoseaug pnosesep pnoseoct pnosenov pnosedec iactiv12 hfeverev rashev rash12  sitesev rashage rclear12 rawake12 eczemaev brthev brth12 brth1m exbrthev  exbrth12 exbrth1m wwokenev wwoken12 wwoken1m cwokenev cwoken12 cwoken1m  sabrthev sabrth12 sabrth1m dschool dsex dwhezev dwhez12 dnwhez12 dawake12  dspeech12 dexwhez12 c1 c2 c99;  MISSING=.;  CLUSTER = dschool;  USEVAR=dnwhez12 dawake12 dspeech12 dexwhez12 c2 c99 dsex;  WITHIN = dsex;  BETWEEN = c2 c99;  CATEGORICAL = dnwhez12 dawake12 dspeech12 dexwhez12;  ANALYSIS: type = twolevel; estimator = wlsmv;  MODEL:  %WITHIN%  whzw BY dnwhez12* dawake12* dspeech12* dexwhez12*;  whzw @1;  whzw ON dsex;  %BETWEEN%  dnwhez12 WITH dawake12 dspeech12 dexwhez12;  dawake12 WITH dspeech12 dexwhez12;  dspeech12 WITH dexwhez12;  dnwhez12 dawake12 dspeech12 dexwhez12 ON c2 c99;  OUTPUT: sampstat cinterval residual stand(stdy);  PLOT: type = plot1 plot2 plot3; |
