# Supplementary material for: A new model of wheezing severity in young children using the validated ISAAC wheezing module: A latent variable approach with validation in independent cohorts
Source: PLoS One. 2018 Apr 17;13(4):e0194739. doi: 10.1371/journal.pone.0194739 (PMC5903664; doi:10.1371/journal.pone.0194739)
Supplement: S3 Appendix — (DOCX) [file pone.0194739.s005.docx]

S3 Appendix: Cohort 3 Analyses

Table of Contents

[Contents 2](#_Toc503002928)

[Variable key 2](#_Toc503002929)

[Model 3.1 2](#_Toc503002930)

[m3.1: lavaan code 2](#_Toc503002931)

[m3.1: Sample statistics (observed covariance matrix and thresholds) 2](#_Toc503002932)

[m3.1: Model implied covariance matrix 3](#_Toc503002933)

[m3.1: Residual correlation matrix for Model 3.1 3](#_Toc503002934)

[Model 3.2 3](#_Toc503002935)

[m3.2: lavaan code 3](#_Toc503002936)

[m3.2: Sample statistics (observed covariance matrix and thresholds) 4](#_Toc503002937)

[m3.2: Model implied covariance matrix 4](#_Toc503002938)

[m3.2: Residual correlation matrix for Model 3.2 4](#_Toc503002939)

[Model 3.3 5](#_Toc503002940)

[m3.3: lavaan code 5](#_Toc503002941)

[m3.3: Sample statistics (observed covariance matrix and thresholds) 5](#_Toc503002942)

[m3.3: Model implied covariance matrix 6](#_Toc503002943)

[m3.3: Residual correlation matrix for Model 3.3 6](#_Toc503002944)

[Model 3.4 6](#_Toc503002945)

[m3.4: lavaan code 6](#_Toc503002946)

[m3.4: Sample statistics (observed covariance matrix and thresholds) 6](#_Toc503002947)

[m3.4: Model implied covariance matrix 7](#_Toc503002948)

[m3.4: Residual correlation matrix for Model 3.4 7](#_Toc503002949)

[Model 3.5 7](#_Toc503002950)

[m3.5: lavaan code 7](#_Toc503002951)

[m3.5: Sample statistics (observed covariance matrix and thresholds) 8](#_Toc503002952)

[m3.5: Model implied covariance matrix 8](#_Toc503002953)

[m3.5: Residual correlation matrix for model 1.5 9](#_Toc503002954)

# Contents

This file provides supplemental modeling information for the Cohort 3 analyses. We provide *lavaan* code for the primary Cohort 3 models (see Table 4 in the primary text). Additionally, we provide observed and model-implied covariance marixes for each model, as well as the residual correlation matrix.

# Variable key

Below we define the observed variables used in the analyses. For detailed information about *lavaan* modeling code, see the [lavaan website](http://lavaan.ugent.be/).

- dnwhez12 = ISAAC-WM item from the one-year follow-up assessment asking about the frequency of wheezing attacks in the past year (4-level ordinal variable)
- dawake12 = ISAAC-WM item from the one-year follow-up assessment asking about the frequency of wheeze-induced sleep disturbance in the past year (3-level ordinal variable)
- dspeech12 = ISAAC-WM item from the one-year follow-up assessment asking about the frequency of wheeze-induced speech disturbance in the past year (2-level ordinal variable)
- dexwhez12 = ISAAC-WM item from the one-year follow-up assessment asking whether the child has experienced exercise-induced wheeze

# Model 3.1

## m3.1: lavaan code

m3.1.syn <- '
whzsev =~ dnwhez + dawake + dspeech + dexwhez
'

m3.1 <- cfa( m3.1.syn, data = ehaas.wide1, estimator = 'wlsmv', std.lv = T, missing = 'pairwise',
 ordered = c( 'dnwhez','dawake','dspeech','dexwhez' ) )

## m3.1: Sample statistics (observed covariance matrix and thresholds)

## $cov
## dnwhez dawake dspech dexwhz
## dnwhez 1.000
## dawake 0.337 1.000
## dspeech 0.307 0.304 1.000
## dexwhez 0.326 0.114 0.147 1.000
##
## $mean
## dnwhez dawake dspeech dexwhez
## 0 0 0 0
##
## $th
## dnwhez|t1 dnwhez|t2 dnwhez|t3 dawake|t1 dawake|t2 dspeech|t1
## -0.478 0.566 0.981 0.140 0.799 1.221
## dexwhez|t1
## 0.591

## m3.1: Model implied covariance matrix

## dnwhez dawake dspech dexwhz
## dnwhez 1.000
## dawake 0.341 1.000
## dspeech 0.339 0.199 1.000
## dexwhez 0.296 0.174 0.173 1.000

## m3.1: Residual correlation matrix for Model 3.1

## dnwhez dawake dspech dexwhz
## dnwhez 0.000
## dawake -0.003 0.000
## dspeech -0.032 0.106 0.000
## dexwhez 0.030 -0.059 -0.025 0.000

# Model 3.2

## m3.2: lavaan code

m3.2syn <- '
whzsev =~ dnwhez + dawake + dspeech + dexwhez
whzsev ~ white + male
'

m3.2 <- sem( m3.2syn, data = ehaas.wide1, estimator = 'wlsmv', std.lv = T, missing = 'pairwise',
 ordered = c( 'dnwhez','dawake','dspeech','dexwhez') )

## m3.2: Sample statistics (observed covariance matrix and thresholds)

## $res.cov
## dnwhez dawake dspech dexwhz
## dnwhez 1.000
## dawake 0.310 1.000
## dspeech 0.281 0.263 1.000
## dexwhez 0.294 0.069 0.099 1.000
##
## $res.int
## dnwhez dawake dspeech dexwhez
## 0 0 0 0
##
## $res.th
## dnwhez|t1 dnwhez|t2 dnwhez|t3 dawake|t1 dawake|t2 dspeech|t1
## -0.258 0.799 1.223 0.585 1.262 2.004
## dexwhez|t1
## 0.812
##
## $res.slopes
## white male
## dnwhez 0.018 0.317
## dawake 0.118 0.511
## dspeech 0.534 0.452
## dexwhez -0.153 0.485
##
## $cov.x
## white male
## white 0.171
## male -0.003 0.224

## m3.2: Model implied covariance matrix

## dnwhez dawake dspech dexwhz
## dnwhez 1.000
## dawake 0.294 1.000
## dspeech 0.272 0.254 1.000
## dexwhez 0.209 0.196 0.181 1.000

## m3.2: Residual correlation matrix for Model 3.2

## dnwhez dawake dspech dexwhz
## dnwhez 0.000
## dawake 0.016 0.000
## dspeech 0.009 0.009 0.000
## dexwhez 0.084 -0.127 -0.082 0.000

# Model 3.3

## m3.3: lavaan code

m3.3syn <- '
whzsev =~ dnwhez + dawake + dspeech + dexwhez
whzsev ~ white + male
asthmadx.84 ~ whzsev + white + male
'

m3.3 <- cfa( m3.3syn, data = ehaas.wide1, estimator = 'wlsmv', std.lv = T, missing = 'pairwise',
 ordered = c( 'dnwhez','dawake','dspeech','dexwhez','asthmadx.84' ) )

## m3.3: Sample statistics (observed covariance matrix and thresholds)

## $res.cov
## dnwhez dawake dspech dexwhz ast.84
## dnwhez 1.000
## dawake 0.310 1.000
## dspeech 0.281 0.263 1.000
## dexwhez 0.294 0.069 0.099 1.000
## asthmadx.84 0.480 0.098 0.054 0.145 1.000
##
## $res.int
## dnwhez dawake dspeech dexwhez asthmadx.84
## 0 0 0 0 0
##
## $res.th
## dnwhez|t1 dnwhez|t2 dnwhez|t3 dawake|t1 dawake|t2
## -0.258 0.799 1.223 0.585 1.262
## dspeech|t1 dexwhez|t1 asthmadx.84|t1
## 2.004 0.812 0.217
##
## $res.slopes
## white male
## dnwhez 0.018 0.317
## dawake 0.118 0.511
## dspeech 0.534 0.452
## dexwhez -0.153 0.485
## asthmadx.84 -0.240 0.333
##
## $cov.x
## white male
## white 0.171
## male -0.003 0.224

## m3.3: Model implied covariance matrix

## dnwhez dawake dspech dexwhz ast.84
## dnwhez 1.000
## dawake 0.309 1.000
## dspeech 0.286 0.142 1.000
## dexwhez 0.284 0.141 0.130 1.000
## asthmadx.84 0.411 0.204 0.188 0.187 1.000

## m3.3: Residual correlation matrix for Model 3.3

## dnwhez dawake dspech dexwhz ast.84
## dnwhez 0.000
## dawake 0.000 0.000
## dspeech -0.005 0.121 0.000
## dexwhez 0.010 -0.072 -0.031 0.000
## asthmadx.84 0.069 -0.106 -0.134 -0.042 0.000

# Model 3.4

## m3.4: lavaan code

m3.4syn <- '
whzsev =~ dnwhez + dawake + dspeech + dexwhez
whzsev ~ white + male
whzdr12.84 ~ whzsev + white + male
'

m3.4 <- cfa( m3.4syn, data = ehaas.wide1, estimator = 'wlsmv', std.lv = T, missing = 'pairwise',
 ordered = c( 'dnwhez','dawake','dspeech','dexwhez','whzdr12.84' ) )

## m3.4: Sample statistics (observed covariance matrix and thresholds)

## $res.cov
## dnwhez dawake dspech dexwhz w12.84
## dnwhez 1.000
## dawake 0.310 1.000
## dspeech 0.281 0.263 1.000
## dexwhez 0.294 0.069 0.099 1.000
## whzdr12.84 0.483 0.248 0.130 -0.033 1.000
##
## $res.int
## dnwhez dawake dspeech dexwhez whzdr12.84
## 0 0 0 0 0
##
## $res.th
## dnwhez|t1 dnwhez|t2 dnwhez|t3 dawake|t1 dawake|t2
## -0.258 0.799 1.223 0.585 1.262
## dspeech|t1 dexwhez|t1 whzdr12.84|t1
## 2.004 0.812 1.223
##
## $res.slopes
## white male
## dnwhez 0.018 0.317
## dawake 0.118 0.511
## dspeech 0.534 0.452
## dexwhez -0.153 0.485
## whzdr12.84 0.250 0.549
##
## $cov.x
## white male
## white 0.171
## male -0.003 0.224

## m3.4: Model implied covariance matrix

## dnwhez dawake dspech dexwhz w12.84
## dnwhez 1.000
## dawake 0.332 1.000
## dspeech 0.290 0.189 1.000
## dexwhez 0.205 0.134 0.117 1.000
## whzdr12.84 0.412 0.269 0.235 0.166 1.000

## m3.4: Residual correlation matrix for Model 3.4

## dnwhez dawake dspech dexwhz w12.84
## dnwhez 0.000
## dawake -0.023 0.000
## dspeech -0.009 0.074 0.000
## dexwhez 0.089 -0.065 -0.018 0.000
## whzdr12.84 0.071 -0.021 -0.104 -0.199 0.000

# Model 3.5

## m3.5: lavaan code

m3.5syn <- '
whzsev =~ dnwhez + dawake + dspeech + dexwhez
whzsev ~ white + male
whzemerg12.84 ~ whzsev + white + male
'

m3.5 <- cfa( m3.5syn, data = ehaas.wide1, estimator = 'wlsmv', std.lv = T, missing = 'pairwise',
 ordered = c( 'dnwhez','dawake','dspeech','dexwhez','whzemerg12.84' ) )

## m3.5: Sample statistics (observed covariance matrix and thresholds)

## $res.cov
## dnwhez dawake dspech dexwhz w12.84
## dnwhez 1.000
## dawake 0.310 1.000
## dspeech 0.281 0.263 1.000
## dexwhez 0.294 0.069 0.099 1.000
## whzemerg12.84 0.628 0.369 0.304 0.104 1.000
##
## $res.int
## dnwhez dawake dspeech dexwhez whzemerg12.84
## 0 0 0 0 0
##
## $res.th
## dnwhez|t1 dnwhez|t2 dnwhez|t3 dawake|t1
## -0.258 0.799 1.223 0.585
## dawake|t2 dspeech|t1 dexwhez|t1 whzemerg12.84|t1
## 1.262 2.004 0.812 1.169
##
## $res.slopes
## white male
## dnwhez 0.018 0.317
## dawake 0.118 0.511
## dspeech 0.534 0.452
## dexwhez -0.153 0.485
## whzemerg12.84 0.182 0.611
##
## $cov.x
## white male
## white 0.171
## male -0.003 0.224

## m3.5: Model implied covariance matrix

## dnwhez dawake dspech dexwhz w12.84
## dnwhez 1.000
## dawake 0.324 1.000
## dspeech 0.297 0.206 1.000
## dexwhez 0.206 0.143 0.131 1.000
## whzemerg12.84 0.562 0.390 0.357 0.248 1.000

## m3.5: Residual correlation matrix for model 1.5

## dnwhez dawake dspech dexwhz w12.84
## dnwhez 0.000
## dawake -0.014 0.000
## dspeech -0.016 0.057 0.000
## dexwhez 0.088 -0.074 -0.032 0.000
## whzemerg12.84 0.066 -0.021 -0.053 -0.144 0.000
